# An Indicator of the Impact of Climatic Change on European Bird Populations

**DOI:** 10.1371/journal.pone.0004678

**Published:** 2009-03-04

**Authors:** Richard D. Gregory, Stephen G. Willis, Frédéric Jiguet, Petr Voříšek, Alena Klvaňová, Arco van Strien, Brian Huntley, Yvonne C. Collingham, Denis Couvet, Rhys E. Green

**Affiliations:** 1 The Royal Society for the Protection of Birds & European Bird Census Council, The Lodge, Sandy, Bedfordshire, United Kingdom; 2 Institute of Ecosystem Science, School of Biological and Biomedical Sciences, Durham University, Durham, United Kingdom; 3 Muséum National d'Histoire Naturelle, UMR 5173 MNHN-CNRS-UPMC, CRBPO, Paris, France; 4 Czech Society for Ornithology, Prague, Czech Republic; 5 Statistics Netherlands, Voorburg, the Netherlands; 6 The Royal Society for the Protection of Birds, The Lodge, Sandy, Bedfordshire, United Kingdom; 7 Conservation Science Group, Department of Zoology, University of Cambridge, Cambridge, United Kingdom; University of Kent, United Kingdom

## Abstract

Rapid climatic change poses a threat to global biodiversity. There is extensive evidence that recent climatic change has affected animal and plant populations, but no indicators exist that summarise impacts over many species and large areas. We use data on long-term population trends of European birds to develop such an indicator. We find a significant relationship between interspecific variation in population trend and the change in potential range extent between the late 20^th^ and late 21^st^ centuries, forecasted by climatic envelope models. Our indicator measures divergence in population trend between bird species predicted by climatic envelope models to be favourably affected by climatic change and those adversely affected. The indicator shows a rapid increase in the past twenty years, coinciding with a period of rapid warming.

## Introduction

Evidence is accumulating that climatic change in recent decades [1] has altered many biological phenomena across the globe, including the geographical ranges and abundance of plants and animals [2], [3], and the timing of events in their lives such as growth, reproduction and migration [4], [5]. Scientists and policy makers are calling for the development of indicators of the impacts of climatic change on biodiversity based upon these phenomena [6], [7]. The purpose of such indicators is to summarise sets of related impacts, to describe how they are changing in an accessible way, to raise awareness of the biological consequences of climatic warming, and to assist both in setting targets for the reduction of impacts and in guiding the implementation of mitigation and adaptation measures [8]. However, inadequate data, insufficiently validated models and the considerable uncertainty that remains regarding climatic change itself, and its consequences for species and populations [9], [10], have impeded the identification of suitable indicators and hence progress in the policy arena.

Here, we make practical progress by developing a biological indicator of climatic change impacts in two steps. First, we test the performance of projections of change in the extent of species' geographical range (CLIM, based upon climatic envelope models) as predictors of observed interspecific variation in long-term change in population size of land bird species over a large part of Europe. Testing the performance of climatic envelope models is necessary to address concerns about their accuracy in predicting species' future responses to climatic change [11]–[14]. Because our response variable is a measure of the change in size of the breeding population in a large part of Europe, we would ideally use model projections of the effect of climatic change on population size. However, models capable of this are not sufficiently developed, so we instead used model projections of change in range extent. Following Brown [15], we suggest that both determinants of a species' population size, geographic range and local density, are affected in parallel ways by changes in the physical and biotic variables that reflect species' requirements. Hence, we propose that CLIM, a projection of change in potential range extent, can act as a proxy for changes in the suitability of the climate for a given species. We expect species to respond by increasing or decreasing in density within their existing range, by expanding or contracting the extent of their range, or by a combination of both. In their present form, our data on population changes do not allow us to separate observed population change into changes in local abundance or in geographic range. On this basis, if climatic change has already started to be a driver of bird population changes in Europe, we expect a positive correlation between observed change in abundance and CLIM. Having found a robust relationship of this kind, our second step is to construct a climatic impact indicator based upon the divergence in population trends between species expected to be positively and negatively affected by climatic change.

### Analysis

The analysis was able to draw on European trends for all 124 species adequately covered by the Pan-European Common Bird Monitoring Scheme (Text S1). Of these, we excluded two species of raptors from the calculation of the indicator because their numbers, trends and realized geographical ranges have been heavily influenced historically by pesticide poisoning and human persecution. We excluded a further fourteen species from a comparison of observed population trends for 1980–2005 with climatic envelope model projections and retrodictions because trend information was only available after 1990 (Text S1). Hence, for our test of the performance of climatic envelope models, we used long-term population trends based upon annual indices of the population size of 108 bird species in 20 European countries during the period 1980–2005 (Text S1, Tables S1 and S2). The trend of the combined population of each species in this set of countries was calculated as the regression coefficient of annual counts on calendar year from a log-linear Poisson regression model [8]. Population series extended to 2005 for nearly all species and countries, but began at different times. National and supranational trends were calculated from these in a hierarchical fashion using a model (applied in the software package TRIM) in a way that allows for missing observations [16], [17]. We first calculated national species' trends and combined them in four regional groupings (Text S1). Any missing year totals were then estimated from other countries in the same region on the assumption that those countries shared similar population changes being subject to similar environmental pressures. The combined species' trends were weighted to allow for the fact that different countries hold different proportions of the European population. Having estimated regional trends, these were then combined to generate European indices for each species [16, Text S1]. We calculated values of CLIM using climatic envelope models fitted to the European breeding season distribution of each bird species mapped during the 1980s [18]. The model describes each species' distribution in relation to 1961–1990 means of three bioclimate variables; the annual sum of positive differences between the daily mean temperature and 5°C (in °C days); mean temperature of the coldest month (°C); and an estimate of the annual ratio of actual to potential evapotranspiration [8]. We simulated the extent of the recent and potential future geographical range of each species within the combined area of the 20 countries for which population trend data were available using the climatic envelope model and the observed 1961–1990 means of the bioclimate variables and means of these variables projected for 2070–2099 by a General Circulation Model (GCM) and Special Report on Emissions Scenario (SRES) emissions scenario [19]. The CLIM value for a species is the log of the ratio of the extent of the future potential range to that of the recent simulated range. We had a clear expectation of the direction of the effect of the CLIM, and other climate response predictors, on long-term population trends (Table S3). To test for sensitivity of our results to our choice of GCM and SRES scenario, we combined results from three GCMs (HadCM3, Echam4 and GFDL) with two SRES scenarios (A2 and B2), to give six variants of CLIM (CLIMHaA2, CLIMHaB2, CLIMEcA2, CLIMEcB2, CLIMGfA2 and CLIMGfB2: [8]). We also calculated the average of these six values to create an ensemble forecast (CLIMEns). It has been suggested that an ensemble of projected species' range changes should be used, based upon a range of different climatic envelope model fitting procedures [12]. However, we chose to use a single robust modelling procedure and a small set of independent variables, which are the same for every species, for simplicity and because this procedure performed well both in predicting static distribution data for parts of Europe other than those used to generate the models [20], [21], and in predicting interspecific variation in change of abundance ([22] and see below).

Variation among species of European and other birds in the rate of recent population change has been reported to be correlated with anthropogenic and non-anthropogenic factors, other than climatic change, associated with their breeding habitat [16], migratory behaviour [23] and life history characteristics (for which we use body mass as a proxy) [24]. For this reason, we examined the relationship of population trend to CLIM, both on its own and against the background of these potentially confounding variables, using model averaging [25].

**Figure 1:**
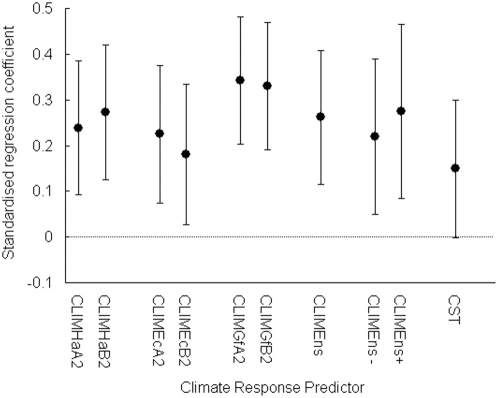
The relationship of interspecific variation in recent population trends (1980–2005) of 108 European land bird species to projections of potential future geographical range change (CLIM) and retrodictions of climate suitability trend (CST) for observed recent climate (1980–2002), both derived from climatic envelope models fitted to the observed European geographical range of each species in the 1980s. The figure shows the standardised regression coefficient of population trend on each variable, with 90% confidence intervals, derived from model averaging of multiple regression models which also take into account the effects of body mass, habitat and migratory behaviour (Table S9). Positive coefficients indicate a positive relation between population trend and CLIM or CST; coefficients with confidence intervals that do not overlap zero are statistically significant.

We found a highly significant positive correlation between interspecific variation in recent population trend and CLIM (Figure 1, Text S2, Figure S3). Population trend also covaried significantly with breeding habitat and migratory behaviour and, less consistently, with mean body mass, but there were negligible effects on the relationship of population trend to CLIM of including these variables in regression models (Figure 1, Text S1, Text S2, Table S4). Neither was there evidence that these variables affected the slope of the relationship between population trend and CLIM (Text S1, Text S2). Population trend also correlated significantly with CLIM after allowing for phylogenetic relationships among species (Text S1, Table S5). The variants of CLIM derived from different GCM/SRES combinations were strongly correlated with one another (Table S6) and the differences among them in the strength of their relationship to population trend were small (Figure 1). Previous studies have suggested more robust evidence for changes in the distribution and abundance of species with expected positive, rather than negative, effects of climatic change [26], [27]. However, we found no consistent difference in the strength of the relationship between population trend and CLIM variables for these two groups of species when considered separately (Figure 1 and Table S7).

An assumption of our use of CLIM is that bioclimate variables have already changed since 1980 in the direction of the GCM projections of change between 1961–1990 and 2070–2099. We tested this by examining the correlation across bird species between CLIM and the recent trend in the suitability of the climate within our study area, based upon observed climatic change during the period when bird populations were monitored. We used the climatic envelope models, described above, and annual values of the bioclimate variables to calculate the probability of occurrence of each species in each of the years 1980–2002 for every 50-km UTM square in the study area. The probabilities for each year were then averaged across all squares to obtain annual mean probability of occurrence, and ordinary least squares linear regression was used to calculate the slope of the regression of logit annual mean probability of occurrence on year. We refer to this slope as the species' *climate suitability trend* (CST). As expected if bioclimate variables have already changed since 1980 in the direction of the GCM projections, there was a highly significant relationship between interspecific variation in CLIMEns and that in CST (*r* = 0.601, *P*<0.0001; range of *r* for the 6 CLIM variants, 0.523–0.628). As a final test of the performance of the climatic envelope models, we examined the relationship between observed population trend and CST. There was a marginally significant positive correlation between observed population trend and CST when effects of potentially confounding variables were allowed for using model averaging (Fig. 1, *z* = 1.64, *P* = 0.050), though the relationship was non-significant when these other effects were ignored (Tables S4 and S5).

The significant positive correlation between observed changes of population and projections of change in potential geographical range derived from climatic envelope models provides support for the use of these models to derive a climatic impact indicator. Our second step was therefore to construct such an indicator from the observed population trajectories of 122 bird species with data available for any part of the period 1980–2005 (Text S1, Table S1). Here we were able to make use of all the reliable trend data available, excluding data for two raptor species whose populations have been strongly affected by man (Text S1). We divided these species into those for which the climatic envelope model projection indicated an increase in potential geographical range (CLIMEns+) and those with projected decreases in geographical range (CLIMEns−). Future potential range was smaller than the recent simulated range (CLIMEns<0) for 75% of species (range for the six GCM/SRES scenarios separately: 61–79%) [20], [21]. For each of the two groups of species, we calculated a multi-species population index from population indices for individual species, with the weight of the contribution of each species to the index being its absolute value of CLIMEns. Hence, population trends of species predicted by our models to be more strongly affected by climatic change (either positively or negatively) would have greater influence on the direction of the composite trends in the multi-species index. The following calculation was performed separately for each group. For each species, we had a time series of population index values, some of which were complete (data for all the years 1980–2005) and some of which had no index values for the early years. We converted the series for the *i*th species, of length *k*, into *k*-1 values of *X_i,j_* = log (*I_i,j+1_ / I_i,j_*), where *I_i,j_* is the population index value for the year *j* and *I_i,j_*_+1_ is the population index in the following year. We calculated a weight *w_i,j_* for the *i*th species in the *j*th year as
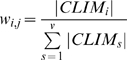
(1)where *v* is the number of species for which there is an eligible value of *X_i,j_* in the *j*th year, any species for which no value could be calculated being excluded in that year. We then calculated the sum of *w_i,j_·X_i,j_* across species for the *j*th year. This represents the log of the proportional change in the index between year *j* and year *j*+1 for this group of birds. Setting the initial value of the index to 100 in 1980, we then used these change values to calculate successive values of the index for all the years in the series.

The indices for the two groups of species do not in themselves provide an indicator of the impact of climatic change upon bird population trends. If both groups are similarly susceptible to other environmental changes, such as agricultural intensification or habitat loss, then they might both be declining at similar rates if there was no effect of climatic change. However, it would be expected that the group of species expected to be positively affected by climatic change (CLIMEns+) would decline less rapidly than those negatively affected (CLIMEns−) during a period when climatic change was occurring in the direction projected for the long term by GCMs. Hence, the impact of climatic changes (both positive and negative) on bird populations can then be summarised in a single indicator, which we term the Climatic Impact Indicator (CII). This is calculated in a given year as the ratio of the index for CLIMEns+ species to that for CLIMEns− species, and has confidence limits obtained using a bootstrap method [8].

**Figure 2:**
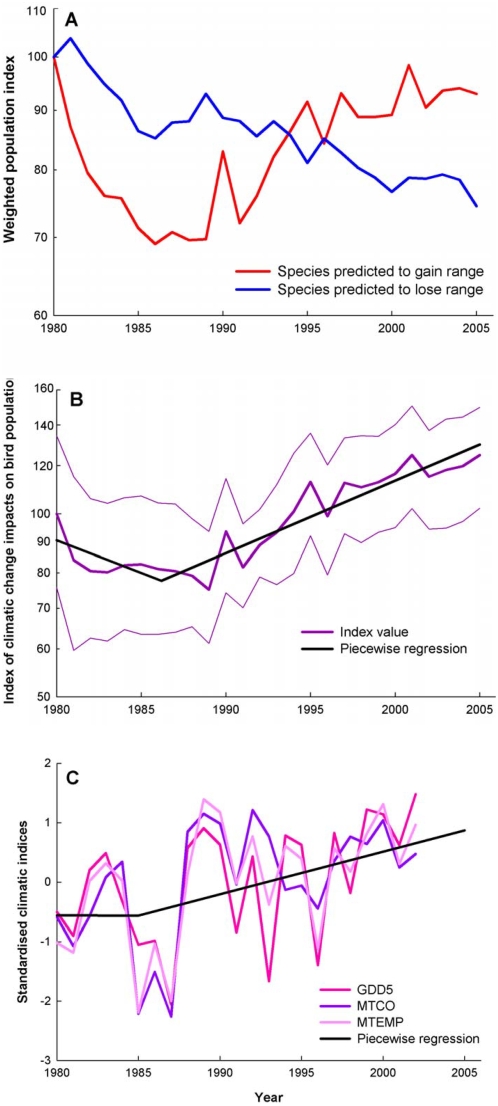
Indices of the impact of climatic change on populations of European birds, 1980–2005, and of climatic change in Europe. (A) Weighted composite population trajectories of two groups of widespread European land birds from 1980 to 2005. The indices are set to 100 in 1980. The red line shows the weighted composite trend of 30 bird species expected, from climatic envelope models, to increase their geographical range in the study region under projected climatic change, the blue line shows the trend of 92 species expected to lose range under projected climatic change. Potential range change projections were averaged over three GCMs and two emissions scenarios. (B) The Climatic Impact Indicator (CII) (magenta line), which is the ratio of the index for species whose potential geographical ranges are expected to expand to that for those expected to contract because of climatic change. The indicator is set to 100 in 1980. Thin lines show 90% bootstrap confidence intervals for annual values from 10,000 bootstrap replicates. The black line shows a piecewise least squares regression model fitted to the annual values (Table S10). A randomisation test (10,000 randomisations) indicates a probability of 0.047 of obtaining as positive or more positive a linear trend as that from the regression of log CII on year over the whole period (supporting online text). (C) Changes in three measures of climate in the countries from which bird data were collected: MTEMP – mean annual temperature (pink); MTCO – mean temperature of the coldest month (blue); and GDD5 – annual temperature sum above 5°C (red), each standardised to have zero mean and unit variance. The black line shows piecewise least squares regression fitted to the annual standardised values for all three variables (Table S10).

The multi-species population indices for CLIMEns+ and CLIMEns− species groups both declined in the early 1980s, but from the latter part of that decade onwards, the CLIMEns+ index (30 species) increased, whilst the CLIMEns- index (92 species) continued to decline (Figure 2A). The CII, reflecting the divergence of the indices for the two groups, declined non-significantly in the early 1980s, but has shown a roughly linear increase from then onwards (Figure 2B). Equivalent group indices and CII values, calculated separately for each of the component GCM/SRES scenarios, show a similar pattern (Text S1, Text S2, Figure S1). Adjustment of the indices and indicator to allow for possible effects of breeding habitat, migratory behaviour and mean body mass only alters this pattern very slightly (Text S2, Figure S2, Table S11), so it is not spuriously driven by the other potential sources of environmental change that we examined. To put the changes in the CII into the context of observed climatic change, we show observed changes in annual mean temperature and bioclimate variables reflecting winter and summer warmth for the countries in which the birds were monitored (available to 2002, Figure 2C, Text S2, Figure S4). The temporal pattern of change of the CII resembles that for temperature. The CII declined and temperature was stable until the late 1980s, after which both increased (Figure 2B and 2C). The stable temperatures in the early 1980s represent the end of a period of approximately stable annual average temperature in Europe that began around 1950 [28].

## Discussion

Concerns have been expressed about the reliability of climatic envelope models as tools to predict species' future responses to climatic change [11]–[14]. We suggest that the most appropriate tests of the utility of climatic envelope models are of two types. First, comparison of observed range with the simulated potential range in cases where the model used for the simulation was fitted using data from a different area than that from which the test observations came. Tests of this kind are described in the Supplementary Information and indicate good model performance. However, because this approach does not fully eliminate effects of spatial autocorrelation, a second, more stringent approach is also required. This is to compare observed changes in population or range with model projections of range change. Our tests of the performance of climatic envelope models in this regard reveal that variation in population trend among European bird species is significantly correlated with model projections of change in the extent of the species' potential geographical range associated with future climatic change. A further potential concern arises when climatic envelope models are fitted to only part of the entire geographical range of a species. This is the case in our study where more than half of the bird species had part of their breeding range in North Africa, an area not covered by the atlas data we used to fit the climatic envelope models. This could lead to unreliable projections of future range change, but this does not appear to be the case as the correlations between population trend and the CLIM variables do not differ between those species with part of their breeding range in North Africa compared to those that do not (Text S2, Table S12).

In addition, population trends showed a near-significant positive correlation with retrodicted trends in the suitability of the climate for each species (CST), based upon the climatic envelope models and observed recent climatic change. This result parallels that of a similar study of recent population trends of rare breeding birds in the United Kingdom, which found that interspecific variation in observed population trends was correlated with retrodictions of CST using climatic data and the same climatic envelope models as those used in the present study (22). We take these findings to indicate that retrodictions and projections based upon these climatic envelope models are useful in predicting observed changes in bird populations.

The weak relationship between observed changes in abundance and trends in the retrodicted suitability of the climate for each species (CST), contrasts with the highly significant relationships between observed population trend and longer-term projections of change in potential range for each species (the CLIM variants). As calculated, CST is sensitive to extreme annual values of meteorological variables and often has relatively low precision as a result. This might cause a poor correlation of CST with bird trends if bird species' population responses smooth out the effects of such short-term extreme fluctuations to a greater extent than the statistical procedure used to fit the CST regressions. In contrast, the CLIM projection may represent a more strongly smoothed version of the climate suitability trend because it is calculated from climatic change projected over a much longer period.

Our results indicate that climatic change is already having a detectable continent-wide effect at the level of a large species assemblage, including evidence of negative as well as positive effects (Text S3). We have used these results to justify the development of an indicator of the impact of climatic change on bird populations. The indicator is relevant to policy makers primarily because it can be used to track biological impacts on an annual basis and inform decision-making about policy responses. Slowing the rate of increase of our indicator might be a policy objective, although such a target must recognise inherent time lags in the system. The indicator could continue to be calculated using data from European bird monitoring schemes, and its geographical scope could be extended as new schemes are initiated. It would also be possible to construct separate indicators for individual countries, and for relevant ecological groups of birds. We recognise that our indicator is based upon a subset of species drawn from a single taxon and therefore on its own does not fulfil all the requirements for an appropriate suite of indicators. However, we used crude information on recent population changes for a wider range of bird species than those included in the calculation of the indicator, to demonstrate that the positive relationship between observed and projected trends, upon which the indicator depends, extends well beyond the indicator set to a group of the more abundant bird species in Europe comprising 62% of the total (Text S1, S2). We also show that the indicator set of species provides good coverage of several threat categories, though it clearly under-represents those in the most threatened classes (Text S2, Table S8). The species used in the indicator are widely distributed across European regions and biomes; they include northern and southern European species and those with relatively large, as well as those with relatively restricted, European geographical ranges (see Table S1). We hope that the extension of bird monitoring schemes to a wider group of species and countries will increase coverage over time. It might also be possible to construct separate indicators of impacts of climatic change for plants and other groups of animals. However, the restricted availability of mapped distributions for climatic envelope modelling and of long-term population monitoring data may restrict the scope for such developments in the immediate future.

Although climatic change is believed to be among the most powerful factors shaping future biodiversity in Europe [29], systematic monitoring of impacts is not currently recognised within the established suites of indicators [6]–[8]. For this reason, we hope that our indicator will stimulate similar initiatives.

## Supporting Information

Text S1 Material and Methods
(0.09 MB DOC)

Text S2 Supplementary results
(0.04 MB DOC)

Text S3 Synthesis
(0.04 MB DOC)

Figure S1 Indicators of the impact of climate change derived from each of the six GCM/emissions scenarios and that based upon their average (CLIMEns). (A) The indicator for bird species predicted to gain potential geographical range under climate change for the 6 scenarios and for their average. (B) The indicator for bird species predicted to lose potential geographical range under climate change for the 6 scenarios and for their average. (C) The Climate Impact Indicator, which is the ratio of the indicator in (A) to that in (B). All indicators are set at 100 in 1980 and are plotted on a logarithmic scale.
(6.54 MB TIF)

Figure S2 The Climate Impact Indicator with (blue) and without (red) adjustment for the effects upon population trend of body mass, breeding habitat and migratory status. Thin lines show 90% bootstrap confidence intervals for annual values from 10,000 bootstrap replicates with (blue) and without (red) adjustment, as described above.
(0.20 MB TIF)

Figure S3 Population trends versus climatic projection. Population trends of European birds species (n = 108) plotted against the log ratio of future: recent extent of the potential geographical range obtained using climate envelope models based upon six climate change scenarios and upon their average (CLIMEns).
(0.21 MB TIF)

Figure S4 Standardised values of the bioclimate variables. (A) GDD5 - growing days above 5°C, (B) MTCO - the mean temperature of the coldest month, and (C) MTEMP - the mean annual temperature, calculated from the respective means for all 20 countries (blue) and from an anova model taking into account only conditions in the years in which countries contributed bird population survey data (red).
(4.93 MB TIF)

Table S1 Bird species data.
(0.26 MB DOC)

Table S2 Countries providing data for the PECBMS scheme and the period of monitoring used for analysis in each country.
(0.02 MB DOC)

Table S3 Expected mechanism and direction of effects of Climate Response Predictors (CRPs) on long-term trends in European breeding bird populations.
(0.02 MB DOC)

Table S4 Results of OLS regression of long-term population trend on CRPs.
(0.04 MB DOC)

Table S5 Relationships between European bird species' trends, CRPs and body mass, controlling for the effects of phylogeny using a method of independent contrasts.
(0.03 MB DOC)

Table S6 Interspecific Pearson correlation coefficients among CRPs.
(0.03 MB DOC)

Table S7 Comparison of separate regressions of population trend on CLIM variables for species with negative values of the CLIM variable (CLIM−) and those with positive values of the CLIM variable (CLIM+).
(0.04 MB DOC)

Table S8 Comparison of the European treat status of all breeding species (n = 526) and those used in the indicator analyses above (n = 122).
(0.02 MB DOC)

Table S9 AICc weights for multiple regression models of population trend on CRP variables.
(0.07 MB DOC)

Table S10 Estimates of parameters of two-period piecewise ordinary least squares regression models relating annual values of dependent variables to time (calendar year A.D.).
(0.02 MB DOC)

Table S11 Tests of whether the slope of the relationship between population trend and CLIMEns varies significantly among classes of breeding habitat (HAB) or migratory status (MIG), with log body mass (LMS), or with the goodness-of-fit of the climate envelope model used to calculate CLIMEns (AUC).
(0.02 MB DOC)

Table S12 Comparison of separate regressions of population trend on CRPs for species with or without part of their breeding range in North Africa.
(0.03 MB DOC)
