# Supplementary material for: An Indicator of the Impact of Climatic Change on European Bird Populations
Source: PLoS One. 2009 Mar 4;4(3):e4678. doi: 10.1371/journal.pone.0004678 (PMC2649536; doi:10.1371/journal.pone.0004678)
Supplement: Text S1 — Material and Methods (0.09 MB DOC) [file pone.0004678.s001.doc]

# Text S1. Material and Methods

**The policy relevance and use of indicators**

An indicator in the context of this paper is a group of species whose weighted population trends, when taken together, reflects the average behaviour of the constituent species’ group in relation to climatic change, but might also cast light on trends in attributes of other taxa linked to this environmental driver. Indicators are designed to quantify and communicate complex phenomena in a simple manner, often to multiple audiences [1,2]. A key purpose is to help decision makers formulate policy and then to continue to review adaptive policy and management actions in response to changes in the indicator. Indicators represent a communication bridge between scientists and policy/decision makers. While indicator outputs and presentation needs to be simple and transparent, the methods that underlie indices are often statistically complex (the high level and familiar economic indicators being a prime example). These surrogate measures are frequently used as a proxy for ecosystem function and health because of the complexity, cost and difficulty of measuring these processes directly and precisely. Such indicators are often used in research and wildlife management as diagnostic tools to gain specific knowledge about the environment, but they can also play a vital role at a higher strategic level [1,2]. Of course, high-level indices of this sort are not a substitute for the detailed scientific research and knowledge needed to understand and remedy the causes of change in individual species or ecosystems.

For indicators to be effective they must meet a number of competing scientific and practical criteria, and these include qualities such as scientific credibility, sensitivity to environmental change, links to drivers, clarity of message, relative affordability and ease of update [1-3]. The Climatic Impact Indicator (CII) we propose in this paper scores highly on each of these criteria.

The CII is a ‘pressure’ indicator in the Pressure-State-Response, or Driving force-Pressure-State-Impacts-Response models, commonly used to structure indicator sets (http://www.parliament.uk/documents/upload/postpn312.pdf). It is an indicator of climatic impacts on bird populations in that, as it increases in value, it demonstrates an increasing conformity between variation among species in observed bird population changes and the interspecific variation expected from climate envelope models of the species’ future potential geographical ranges. When the CII is level, this indicates that climatic change is not having any further effect, beyond that which may already have occurred, in the manner predicted by our models. Although other pressures in the environment may be causing populations to decline or increase, the pattern of change when CII is stable is unrelated to that expected from the climate envelope models. Stability in the CII might arise if the climate is not showing any systematic trend over time. The parallel we demonstrate between the change in trend in the CII in the mid-1980s and the change in trend in temperature in Europe is striking (Fig. 2c). The CII is unlike the Living Planet and Wild Bird indicators [1,2], since these measures the state of biodiversity (population size): if populations decline so does the indicator. By contrast, CII is an indicator of the impact of climatic change: as putative effects of climatic change on population size, both positive and negative, proceed, so the indicator goes up.

The proposed indicator is relevant to policy makers because it can be used to track the biological impacts of climatic warming in near real-time, to set targets for the future level of the indicator or its rate of change, and to relate the rate of change in an element of biodiversity to observed temperature change and drivers. Slowing the rate of increase of the indicator, or stabilizing its level, might be suitable policy objectives. Such an indicator would sit among a wider set of environmental indicators linked directly to climatic change (e.g. changes in greenhouse gas emissions, transport volumes, energy intensity, sources of electricity generation), to allow decision makers to establish linkages and integrate responses. Adoption of an indicator and target would have a complex and cascading effect on policy responses (informing measurement, adaptation and mitigation) internationally and nationally, and the indicator would play several complementary roles ranging from raising awareness of biodiversity impacts of climatic change through to informing specific adaptation and mitigation measures. As an indicator, it may provide a broader indication of climatic change impacts on European wildlife and thereby provide a focus for debate on climatic change and biodiversity impacts. The indicator would help to raise awareness and stimulate debate on the threats posed by climatic change to global biodiversity and ecosystem services more broadly.

At the Johannesburg World Summit on Sustainable Development in 2002, world leaders pledged ‘*to achieve by 2010 a significant reduction of the current rate of biodiversity loss at the global, regional and national level as a contribution to poverty alleviation and to the benefit of all life on Earth’*. Consequently, the UN Convention on Biological Diversity (CBD) has established a framework to underpin the target that comprises seven focal areas and twenty-two separate indicators (http://www.cbd.int/2010-target/framework/indicators.shtml). However, the area ‘Threats to Biodiversity’ does not include measures relating to the impacts of climatic change, although there are urgent calls for such indicators from scientists and policy makers [4,5]. Our work provides a practical way to fill this gap by introducing a scientifically robust indicator of climatic change impacts, which we anticipate, in time, will be refined and expanded to capture further dimensions of biodiversity change and climatic impacts.

**Selection of bird species and calculation of European population trends**

We used information on the trends of individual widespread bird species derived from annual breeding bird monitoring schemes in 20 European countries (Table S2), collated by the Pan-European Common Bird Monitoring Scheme (PECBMS) [6]. The PECBMS collates population trend information on as many bird species as possible. Out of about 520 bird species regularly breeding in Europe, we received national trend data on 224 species, from which we were able to calculate reliable European species’ indices for 124 species. National and supranational trends were calculated in a hierarchical fashion using a model (applied in the software package TRIM) [1,7]. TRIM is a program designed to analyse time series of counts with missing observations using Poisson regression (log-linear models [7,8]). The basic model with effects for each site and year is:

log *μ_i,j_*  = *α_i_* + *γ_j_*, (1)

with *α_i_* the effect for site *i* and *γ_j_* the effect for year *j* on the log of expected counts *μ_i,j._*. Missing counts for particular sites were estimated (‘imputed’) from changes in all other sites, or sites with the same characteristics, using covariates. In addition, serial correlation was taken into account. We first calculated national species’ trends and combined them in four regional groupings (West: Ireland, UK, Netherlands, Denmark, Austria, Switzerland, former West Germany, Belgium; North: Sweden, Finland, Norway; East/Central: former East Germany, Estonia, Latvia, Poland, Czech Republic, Hungary; South: France, Spain, Portugal, Italy). Any missing year totals were then estimated from other countries in the same region on the assumption that those countries shared similar population changes and were subject to similar environmental pressures. The combined species’ trends were weighted to allow for the fact that different countries hold different proportions of the European population. The yearly scheme totals were first converted into yearly national population sizes, using the latest information on national population sizes from *Birds in Europe* [9]. These population sizes were assumed to reflect the situation in or around the year 2000. A weighting factor was calculated as the national population size divided by the average of the estimated yearly scheme total for 1999–2001. This weighting factor was applied to all other years of the scheme in order to obtain yearly national population sizes for each year. This means a change in a larger national population has greater impact on the overall trend than a change in a smaller population. The alternative, of weighting national population trends equally, makes little sense in this context because changes in small, insignificant populations could dominate and obscure the genuine European trend. Having estimated regional trends, these were then combined to generate European indices for each species. We included only those species with data available from countries hosting, in aggregate, at least 50% of the European population of a species, following Ref [9]. Excluded species were dominated by waterbirds (wildfowl, waders and gulls) and birds of prey (raptors and owls), where the census methods or frequency of counts were often suboptimal for these species and/or data came from a very limited part of their European ranges. In addition, there were a small number of cases where European indices for species meeting the first criterion could not be computed using our methods because of sparse data. As a second quality check on the indices, we then calculated the ratio of national population size estimates [6] to the scheme year totals calculated by our methods, and excluded species where the values were implausibly small or large (< 1 or >100). Finally, we looked in detail at individual species where their trends were statistically uncertain or highly variable through time, excluding those where their indices were judged unreliable given the scarcity and coverage of the count data. Our conservative approach was designed to remove potentially unreliable and unrepresentative European species’ trends.

Of the 124 species meeting the above criteria, we additionally excluded indices for two raptors, common buzzard, *Buteo buteo*, and Eurasian sparrowhawk, *Accipiter nisus*, because their numbers, trends and realized geographical ranges have been particularly heavily influenced by pesticide poisoning in the 1950 – 60s, and continued human persecution in some areas [10]. In many parts of Europe, their populations were in a state of recovery and re-colonisation during the period of our study. We excluded a further 14 species from the regression analysis of long-term trends because reliable indices were only available after 1990. However, we used the data for these species when calculating the multi-species indicator of climate change impact. Hence, the indicator was calculated from data for 122 species and analysis of long-term trends was carried out on 108 species (Table S1) in 20 countries (Table S2).

The methods used by bird population monitoring schemes differed among countries. Methods included spot/territory mapping method, line and point transects. Schemes differed also in how the sample plots were selected, varying from free choice of sampling plots (i.e. fieldworkers select where they count birds) to systematic, stratified random, or random selection of survey plots. Although free choice poses a potential risk of bias, and there has been a move towards more formal sampling strategies through time, it is argued that such changes have not introduced systematic bias in national or European trends [2,11].

Habitat and migratory status of bird species

Each of the 122 bird species was allocated to one of four broad habitat types: forest, farmland, inland wetland or other (Table S1). A species was allocated to a habitat if more than 50% of the population in the countries contributing data to the PECBMS was judged to use a particular habitat during the breeding season. To do this we used a previous classification [12], regional/national analyses and expert opinion (http://www.ebcc.info/index.php?ID=301). Each species was also allocated to one of **three migratory categories** according to information about the predominant migratory behaviour of populations breeding within the countries contributing data to the PECBMS, as mapped by **Ref [13]** (Table S1**). The categories were: *long distance migrants*** – all or most individuals make regular seasonal movements between the breeding range and a non-breeding range that lies outside the countries contributing data to the PECBMS and outside the Palaearctic region; ***partial migrants*** – populations contain substantial migratory and non-migratory elements, migratory populations make regular movements but these take place within the area covered by our population monitoring network; ***residents*** – most individuals are non-migratory, but some individuals may move long distances, although these movements are rather irregular, and always within the area covered by our population monitoring net.

**Calculation of the CLIM variable**

The CLIM variable for a given species was calculated as the log ratio of the simulated potential future geographical range of the species relative to its simulated recent range. CLIM variables were obtained using climatic envelope models fitted to bird presence-absence information for 50-km UTM squares during the breeding seasons of 1985 – 89 from the European Bird Census Council (EBCC) atlas (*11*) for the whole extent of the study area of the atlas. Values of each of three bioclimatic variables, which have been shown previously to be related to the distribution of many Palaeartic species [14-16], were calculated for each UTM square as previously described [14] using meteorological data for the period 1961 – 1990 (supporting online text). A climatic envelope model relating geographical distribution to the three bioclimate variables was fitted for each species using locally-weighted regression as previously described [17,18]. The models provided a good fit to the data (area under the curve – AUC – of a receiver operating characteristic – ROC – plot; mean AUC of the 122 species = 0.967; lowest value = 0.907).

To project potential responses of bird species to climatic change, we used the climatic envelope models to simulate the potential geographical distribution of each species in the two 30-year periods 1961 – 1990 and 2070 – 2099. The climatic data used for the 1961 – 1990 simulation were as described above. The climatic data used for the 2070 – 2099 simulation were obtained from six future climate scenarios developed from transient simulations made using three general circulation models (GCMs) included in the IPCC 2001 synthesis [19] (GFDL_R30_c [CLIMGf], HadCM3 [CLIMHa] and ECHAM4/OPYC3 [CLIMEc]), combined with two IPCC SRES emissions scenarios (A2 and B2) [20]. This combination of six GCMs and emission scenarios was chosen to cover a range of potential future climate and their average, CLIMEns, was taken to represent an ensemble forecast.

The climatic envelope model was used, together with the climatic data, to generate an expected probability of occurrence of the species in each UTM square. Where this value exceeded a specified threshold, the species was simulated as present. The threshold value selected was that which gave the maximum value of the goodness-of-fit statistic *κ* when the observed atlas distribution and the distribution simulated using the 1961 – 1990 climatic data were compared. The same value of the threshold probability was then used to simulate the potential future distribution using the 2070 – 2099 climatic data. The numbers of simulated occupied squares in the 20 countries from which the bird population trend data were taken were obtained from the simulations based upon the 1961 – 1990 and 2070 – 2099 climates and the log-transformed ratio of future:recent squares taken as the CLIM value for that species. We calculated the ensemble version of CLIM, CLIMEns, as the mean of the CLIM values for a species from the 6 GCM/SRES combinations.

Calculation of the bioclimate variables for recent climate data (1961 – 1990)

Monthly mean values for cloud cover, precipitation and temperature from the CRU CL1.0 0.5º 1961 – 90 climatology [21] (http://www.cru.uea.ac.uk/cru/data/hrg/) were interpolated onto the EBCC grid of *ca*. 50 x 50 km Universal Transverse Mercator (UTM) squares adopted by the breeding birds atlas [22] using a simple bilinear interpolation. Bioclimate values used for modelling species’ distributions were then derived from these climate data using the methods of Ref [23]. The bioclimate variables used in the species’ distribution models were: annual temperature sum above 5ºC (GDD5: ºC days); mean temperature of the coldest month (MTCO: °C); and an estimate of the annual ratio of actual to potential evapotranspiration (APET). These variables can serve to limit species’ ranges either directly, or indirectly through effects on vegetation, prey, predators, competitors or diseases.

Calculation of the bioclimate variables for future scenarios (2070 – 99)

General Circulation Model (GCM) simulated future (2070 – 99) and recent (1961 – 90) monthly mean temperature and precipitation values were downloaded from http://www.ipcc-data.org/sres/gcm_data.html for the three GCMs (GFDL_R30_c, HadCM3 and ECHAM4/OPYC3) and two IPCC SRES emissions scenarios (A2 and B2). Anomalies between the simulated future and recent climate were calculated as differences for temperature and as ratios for precipitation. The anomalies were interpolated onto the EBCC grid by means of Laplacian thin-plate spline surfaces fitted to the GCM anomaly values using longitude and latitude as the independent variables [24]. The temperature and precipitation anomalies were combined with the values from the present-day CRU 1961 – 90 climate during the computation of the projected future values of the bioclimate variables. It should be noted that, because of the current level of uncertainty surrounding GCM simulations of cloud cover, present-day cloud cover values were used when calculating the projected future values of the ratio of actual to potential evapotranspiration (APET). Simulations of species’ potential future distributions were then made using the projected future bioclimate variable values.

Validation of climatic envelope models as descriptions of static geographical ranges

For a given species, the climatic envelope model fitted to the EBCC data can be used to give an expected probability of its occurrence, based upon mean values of 1961 – 1990 bioclimate variables, in each 50-km UTM grid square. For those squares with presence or absence data, we evaluated the goodness-of-fit of the climate envelope model by calculating the area under the curve (AUC) for a receiver operating characteristic (ROC) plot of sensitivity against (1 – specificity) for all possible values of threshold probability [25]. Sensitivity is defined as the proportion of true positives correctly predicted and specificity as the proportion of true negatives correctly predicted.

Such goodness-of-fit tests do not constitute adequate validation tests of climatic envelope models if they are performed using the same data that were used to fit the model. To overcome this problem, we also calculated AUC values after applying a jack-knife procedure. All grid squares of a 6° longitude x 8° latitude panel of the UTM grid system were excluded and the climatic envelope model fitted to the remaining data. This model was used to calculate the expected probability of occurrence of the species in the grid squares of the panel whose data were excluded when fitting the model. This procedure was repeated for all panels to give an expected probability of occurrence for every square. The AUC measure of goodness-of-fit was then calculated from the EBCC data and these expected values. We consider that this procedure of leaving out a large panel when fitting the model is superior to the more common practices either of leaving out one grid square in turn, or of using a set of grid squares selected at random, to obtain a training set to fit a model that then is used to predict for the excluded square(s). Such procedures do not use truly independent data for validation because the data used for validation are from squares close to those used to generate the model used for prediction. There is likely to be spatial autocorrelation because of similarity in unmeasured variables between squares used to fit the model and those used to test it because of their proximity. By leaving out a large panel, rather than single or randomly distributed squares, the problem of spatial autocorrelation and lack of independence is substantially reduced.

Similar autocorrelation problems arise when a model fitted to data for one time period are tested on data for the same geographical area obtained at another time when the climate is different [26]. Because some potentially influential unmeasured variables associated with each grid square are likely to have changed only slightly between the two times (e.g. soil type), the data are autocorrelated and a simple test of goodness-of-fit of the data from the second time period to the model fitted to data from the first time period (or vice versa) does not constitute a validation test on independent data. Only tests of whether changes in distribution (losses and gains of range) are predicted by the model fitted just to data from one time period are adequate in this regard.

Results of our jack-knife procedure for 453 species of breeding birds are given elsewhere [27,28]. The mean jack-knife AUC value for the 122 species used in the calculation of the indicators was 0.86 and all of these species had jack-knife AUC values >0.7, indicating that they were “useful”, whilst for 25% of them the AUC value was >0.9 indicating a “high” performance [29]. Hence, the models perform well on this stringent test and are validated to the extent that they provide a good description of the recent geographical distribution of most species when tested using data other than those used to fit the model.

Research based upon the same climate envelope models has demonstrated their ability to retrodict rare species’ population trends in the UK [30]. Twenty-five-year population trends of 42 bird species rare as breeders in the UK, and at the edge of their European ranges, were examined in relation to changes in climatic suitability calculated using climatic envelope models and observed changes in bioclimate variables. A statistically significant positive correlation was found across species between population trend and climate suitability trend after controlling for potential confounding variables [30].

Additional independent validation of the modelling approach we used has previously been reported. A model fitted to the European range of an introduced plant species was shown successfully to simulate the species’ range in its area of origin in eastern Asia [31]. A model fitted to the present European distribution of the Speckled wood butterfly, *Parage aegeria*, and recent climate was used to hindcast the species’ distribution in the first decade of the twentieth century and the simulated range changes shown to be consistent with those observed [32].

Many of the species included in our analysis have geographical ranges that extend beyond Europe to adjacent parts of Eurasia and/or North Africa (see below). Here we ignore the Eurasian and North African component of bird populations in our modelling of European breeding birds because this is not relevant to the predictions and indicators we developed. We are concerned only with assessing how species' European distribution, and indeed how their abundance and range in a sub-set of the countries of Europe, may be affected by climatic change, and to develop an index that can be assessed as an indication of how much impact climatic change is having on birds in the geo-political region of Europe. For this purpose, it is irrelevant whether a species may, or may not, have an expanding range eastwards across northern Eurasia, or southwards to Africa. Huntley et al. [28] have considered the full implications of climatic change for European species, focusing on those that are endemic or near-endemic.

Regression analysis of population trend on CLIM

We used ordinary least squares regression to relate variation among bird species in population trend to CLIM. The dependent variable in these analyses was the slope of the regression of the natural logarithm of population size on calendar year (LTS) for the 108 species with population data extending earlier than 1990. We performed univariate regression analyses in which LTS was regressed on CLIM. We had a clear *a priori* expectation of the direction of the effect of the CLIM (Table S3), so we used one-tailed *t*-tests of the null hypothesis of no effect of CLIM. We also calculated 90% confidence intervals for each regression coefficient, as well as for the derived standardized regression coefficient. There is evidence from other studies that population trends of European birds vary according to breeding habitat and migratory status. Population trend might also vary with life-history characteristics. We wished to establish whether the expected relationships between population trends and CRP were discernable when these other variables were also taken into account. Therefore, we used a four-category classification of breeding habitat (HAB) and a three-category classification of migratory beheviour (MIG) in least squares multiple regression models (Table S1). We wished to avoid including in our models the many different life history variables which could be assessed, so we used the natural logarithm of mean body mass (LMS= natural logarithm of body mass in grams), which is correlated with many of them. Body mass was estimated as the mean of reported male or female body mass in grams or, where this was not available, from unsexed birds, taken from Ref [33]. We fitted multiple regression models that include as independent variables, in addition to CLIM, all 19 possible combinations of the main effects of LMS, HAB and MIG, individually and in combination, and all the two- and three-way interaction terms between the last three variables. Interactions involving CLIM were not included. We then calculated, for each model, log-likelihoods, AICc values and AICc weights, following Ref [34]. We calculated the weighted average multiple regression coefficient of LTS on CLIM, using AICc weights. We also calculated standardized multiple regression coefficients of LTS on CLIM. Regression coefficients of LTS on CLIM were standardized by multiplying the crude coefficient by the standard deviation of CLIM and dividing by the standard deviation of LTS. Results of univariate and model averaged multiple regressions are shown in Table S4. Note that CLIM and its variants were strongly inter-correlated (Table S6).

We also performed univariate and multiple regressions, with model averaging, of population trend on the CLIM variables after first separating species with negative and positive CLIM values. We fitted piecewise regressions in which the slope of the LTS vs CLIM relationship was assumed to change at CLIM = 0. This was implemented by including in the regression model both the CLIM variable and also CLIM*, where CLIM* = 0 if CLIM<0 and CLIM* = CLIM if CLIM ≥ 0. When this is done, the regression coefficient for CLIM* represents the estimated difference in slope between sections of the range of CLIM (CLIM < 0 and CLIM ≥ 0) in a piecewise regression. We took the *t* value for CLIM* as a test of whether regression slopes differed for CLIM- and CLIM+ species. The difference in slope could plausibly have been in either direction, so we used two-tailed significance tests.

Phylogenetic analyses

Analyses were performed across-species (described above) and within-taxa using a method designed to control for the statistical non-independence of species’ data points due to phylogenetic associations [35]. One way to control for the effects of phylogenetic relatedness is to examine relationships between variables within each pair of taxa below a node in a bifurcating phylogeny [36]. Such comparisons are independent of phylogeny because sister taxa are equally related to each other. We used a model (Comparative analysis by independent contrasts, CAIC version 2.6.9 [37]), which applies Felsenstein’s approach to data sets for which only approximate phylogenies are available. We follow the classification of Ref [38] supplemented by Ref [13]. Independent contrasts were obtained using the option ‘crunch’ with the assumption that all branch lengths in the phylogeny were equal. CAIC requires one variable to be declared as the main independent predictor [37] so we ran the program for each variable in turn (note the results were unchanged when we declared body mass as a single independent predictor). We used linear regression through the origin to examine correlations between species’ trends and response variables, and multiple regression through the origin to examine correlations between trends and CLIM, controlling for the effects of body mass (Table S5). CAIC does not allow more complex, weighted analyses of continuous and categorical variables, nor does it allow model averaging, as described above.

Calculation of the Climatic Impact Indicator and Confidence Limits

The calculation of our indicator rests upon an expectation that a projection of expansion (contraction) of potential geographical range is likely to be associated with an increase (decrease) in breeding population size. We calculated the Climatic Impact Indicator (CII) in a given year as the ratio of the index for CLIM+ species to that for CLIM- species. Confidence limits for the CII were obtained using a bootstrap method. For each of the two groups of species (CLIM+ and CLIM-) we drew a bootstrap sample of *n* bird species at random, with replacement, from the *n* species in the group. We then performed the calculations described above to obtain the annual values of the indices for the CLIM+ and CLIM- species. We then calculated the CII in each year from these, took its natural logarithm and then expressed it as a deviation from the mean of the bootstrap log(CII) across all years. From each of these annual values we then subtracted the difference between log(CII) for 1980 and the mean of log(CII) for 1980-2005 for the original observed series to give (Δlog(CII)). We then repeated this bootstrap sampling and estimation procedure 10,000 times. The 90% confidence limits of Δlog(CII) were taken to be defined by the central 9,000 of the ranked bootstrap set of Δlog(CII) estimates for a given year. The bounds of the confidence interval were then back-transformed.

We performed a randomisation test to obtain the statistical significance of the trend in CII during the period 1980 – 2005. To do this we first calculated Δlog(CII) values for the original dataset, as described above and fitted an ordinary least squares linear regression of Δlog(CII) on calendar year. We then shuffled the CLIM values for all species and reallocated them at random to the population data for a given species. We then calculated Δlog(CII) from the randomised data, fitted the regression of Δlog(CII) on calendar year and recorded whether the value of the regression coefficient was as positive or more positive than that obtained from the real data, as described above. We repeated this randomisation procedure 10,000 times and took the proportion of repetitions where the regression coefficient was as positive as or more positive than that observed from the real data as the probability of the observed trend of CII with calendar year having occurred by chance.

As it stands, the calculations of the climatic change indices for CLIM+ and CLIM- species and of the CII do not take into account any differences there might be between CLIM+ and CLIM- species’ groups in body mass, breeding habitat and migratory status. We calculated an adjusted version of the CII to allow for these effects. To do this we used the 19 regression models of population trend LTS in relation to CLIMEns, with all combinations of the additional nuisance variables LMS, HAB and MIG. For each of these models we calculated the expected value of LTS from the observed values of LMS, HAB and MIG and with CLIMEns set to a constant, its average value. This was done for each of the 122 species used to calculate the indicator. A weighted average of these expected deviations was then obtained from the 19 models, using their AICc weights. The weighted average expected deviation for each species was then calculated as a deviation from the mean of these values across all species. We then subtracted the expected deviation for each species from the *X_i,j_* (see above) for all years in the series for that species. The climatic change indices for CLIM+ and CLIM- species and the CII were then calculated from these adjusted *X_i,j_* as described above.

Change in climate during the period 1980 – 2002 in bird survey countries

The CRU TS 2.1 time-series data were downloaded from http://www.cru.uea.ac.uk/cru/data/hrg/. For each year from 1980 – 2002, annual values of mean annual temperature MTEMP and the bioclimate variables MTCO, the mean temperature of the coldest month, and GDD5, annual temperature sum above 5°C, were calculated for the ca. 50 x 50 km UTM squares of the EBCC grid using the same methods as were used to calculate the recent (1961 – 1990 mean) bioclimate values (see above). More recent data (2003 onwards) were not available. Individual country values for each year were calculated as weighted means, weighting the contribution of each EBCC cell according to the area of the grid cell falling within the country.

To evaluate changes in MTEMP, MTCO and GDD5 in the countries contributing data on bird populations, we fitted an ordinary least squares two-factor anova model (COUNTRY x YEAR) to the data on each dependent variable, excluding years for a particular country when it was not contributing bird data to PECBMS. The main effect of YEAR from these models was taken to represent changes over time in the climatic variable. The year effects were standardized to have zero mean and unit variance to allow them to be plotted together in Figure 2C. For comparison, we also calculated simple annual means of the climatic variables across all countries and standardised those in the same way.

Piecewise regression of the climatic change indicator and bioclimate variables on year

To fit a regression model in which the rate of change of the dependent variable *y* with respect to time *t* changed from a value of *b*_1_ during the early part of the time series to *b*_2_ at breakpoint time *t*′ we used piecewise regression. Hence, *y* is given by:

*y = b_0_ + b_1_ t* for *t* < *t*′ (2)

*y = b_0_ + b_1_ t′ + b_2_ (t - t′)* for *t* ≥ *t*′ (3)

We estimated *b_0_*, *b_1_*, *b_2_* and *t′* by numerically minimising the sum of squares of the residuals from the regression model (Table S10). Annual values of the Climatic Impact Index (CII) were log transformed before analysis. Annual values each of the three climatic variables GDD5, MTCO and MTEMP were standardised by subtracting their mean value for 1980 – 2002 and dividing by their standard deviation; the model was then fitted for all three variables together. These regression models were fitted purely for illustrative purposes and significance tests were performed.

**Species representation within the European data set**

The species in our analyses form a subset of all breeding bird species in Europe focused on the relatively abundant and widely distributed species covered by national breeding bird surveys. Naturally, it is important to understand how representative this subset is of all breeding birds and to do so we have carried out a simple comparison of the European threat status (which is derived from the IUCN Red List Criteria) of our species set compared to all breeding bird in Europe following Ref [9]. This system recognises ten separate categories of threat status for individual species (Table S8).

**Assessing the representativeness of the species included in the indicator**

Our indicator is based upon data from about 23% of all European breeding bird species, comprising those of the most abundant and widespread species for which sufficiently reliable count data were available. It is possible that the indicator would show a different trend if the availability of good quality count data extended to all bird species that breed in Europe. We tested this possibility using cruder information for a comparable time period from *Birds in Europe* (BIE2), a Europe-wide survey of bird populations and trends [9]. BIE2 assigned one of eleven trend scores to each species’ population change in each European country during 1990-2000. Trend scores represent the views of national experts on the range of percentage change in population, which probably includes the actual change. For example, score +2 refers to an increase from 1990 to 2000 of between 20% and 29%, whereas score -2 refers to a decrease of between 20% and 29%. We expressed each of the scores as the ratio *T* of the population in 2000 to that in 1990, assuming a value at the midpoint of each score category. For example, *T* values for scores +2 and -2 were taken to be 1.25 and 0.75 respectively. The national co-ordinators of BIE2 assigned trend scores using a wide variety of information. They used quantitative studies, including atlas studies of distribution and series of population counts, such as PECBMS, but also used less rigorous information and opinion. We only used BIE2 trend scores classed by the national co-ordinators as “well-known” (trend accuracy scores 2 and 3). For a given species, we calculated a weighted mean of *T* across all countries with eligible data, using the estimated size of the population of the species in each country in 1990 as a weight. The result is an estimate of the ratio of population size in 2000 to that in 1990 for the aggregated populations of all the countries included in the calculation. BIE2 gives estimates of population size in each country in about 2000, so we divided that estimate by *T*, to get the population estimate for 1990 for weighting purposes.

Our test of the representativeness of the species used in the indicator was to compare the relationship of log (*T*) with CLIMEns, the projected change in simulated range between 1961-1990 and 2070-2099. If the population trends of the indicator species are positively correlated with CLIMEns to a similar extent as those of the non-indicator species, with no marked difference between the two groups in the slope and intercept of the regressions, then this would be evidence that the indicator is representative of European breeding birds in general. For this test, we calculated CLIMEns for each species, as described above, but for the whole of Europe, rather than just for the PECBMS countries. There were eligible climate envelope and BIE2 data for 390 species, which is 75% of all European breeding species.

**Exploring the relationship between population trends and an independent set of Climate Response Predictors based on species’ recent geographical ranges**

In addition to examining the relationship between bird population trends and CLIM (and its variants), we also looked at the performance of four additional Climate Response Predictors (CRPs) independent of those above and reflecting conditions in the species’ recent geographical ranges. We used the EBCC bird distribution data [22] to obtain three simple measures of the temperature characteristics of the observed recent range of each species. To do this we used mean monthly temperatures from March to August [39]. We calculated: (1) the thermal average, TMEAN, as the mean spring–summer temperature of UTM squares from which the species was recorded in Ref [22]; (2) the thermal maximum, TMAX, as the mean of local spring–summer average monthly temperatures for the warmest 5% of occupied squares; (3) thermal minimum, TMIN, as the mean of local spring–summer average monthly temperatures for the coolest 5% of occupied squares [40,41]. We also calculated the mean latitude of squares simulated to be occupied according to the bioclimate model relating the observed EBCC bird distribution to the 1961 – 1990 climate (LAT). As the climate is already changing in the direction projected by the GCMs, we would expect positive relationships between trends in species’ populations and all of these measures, except for latitude, where we predict a negative correlation (Table S3). Populations of species occupying current breeding ranges with relatively high or low recent average temperatures (captured by TMEAN) are expected to increase or decrease in abundance respectively because warming will render the climate more or less favourable. Similarly, populations whose current breeding range extends to areas with relatively high temperatures, or avoid areas with low temperatures (captured by TMAX and TMIN), are expected to increase in number because warming is predicted to have a favourable effect. Finally, populations located at middle latitudes of species whose current breeding range lies predominantly at higher or lower latitudes (captured by LAT) are expected to decrease or increase in abundance respectively in response to warming, which results in a predicted negative relation between latitude and species’ trend.

**References**

1. Gregory RD, Vorisek P, Van Strien A, Meyling AWG, Jiguet F, et al. (2007) Population trends of widespread woodland birds in Europe. Ibis 149: 78-97.

2. Gregory RD, van Strien A, Vorisek P, Meyling AWG, Noble DG, et al. (2005) Developing indicators for European birds. Philosophical Transactions of the Royal Society B-Biological Sciences 360: 269-288.

3. Balmford A, Bennun L, ten Brink B, Cooper D, Cote IM, et al. (2005) The convention on biological diversity's 2010 target. Science 307: 212-213.

4. Mace GM, Baillie JE (2007) The 2010 biodiversity indicators: challenges for science and policy. Conservation Biology 21: 1402-1413.

5. EEA (2007) European Environment Agency Report No. 11. Halting the loss of biodiversity by 2010: proposal for a first set of indicators to monitor progress in Europe.

6. EBCC (2006) Pan European Common Bird Monitoring http://wwwebccinfo/pecbmhtml.

7. Pannekoek J, van Strien AJ (2001) TRIM 3 Manual. TRends and Indices for Monitoring Data. Research paper no. 0102. (Available at www.ebcc.info). CBS Voorburg, The Netherlands: Statistics Netherlands.

8. McCullagh P, Nelder JA (1989) Generalized Linear Models. Second Edition. London: Chapman & Hall.

9. BirdLife-International (2004) Birds in Europe: population estimates, trends and conservation status. Cambridge, UK: BirdLife International.

10. Thompson DBA, Redpath SM, Fielding AH, Marquiss M, Galbraith CA (2003) Birds of prey in a changing environment. Edinburgh: The Stationary Office.

11. Freeman SN, Noble DG, Newson SE, Baillie SR (2007) Modelling population changes using data from different surveys: the Common Birds Census and the Breeding Bird Survey. Bird Study 54: 61-72.

12. Tucker GM, Evans MI (1997) Habitat for birds in Europe. A conservation strategy for the wider environment. Cambridge, UK: BirdLife International.

13. Snow DW, Perrins CM (1998) The birds of the Western Palearctic: Concise edition. Oxford: Oxford University Press.

14. Hill JK, Thomas CD, Fox R, Telfer MG, Willis SG, et al. (2002) Responses of butterflies to 20th century climate warming: implications for future ranges. Proceedings of the Royal Society of London Series B-Biological Sciences 269: 2163-2171.

15. Huntley B, Collingham YC, Green RE, Hilton GM, Rahbek C, et al. (2006) Potential impacts of climatic change upon geographical distributions of birds. Ibis 148: 8-28.

16. Huntley B, Green RE, Collingham YC, Hill JK, Willis SG, et al. (2004) The performance of models relating species geographical distributions to climate is independent of trophic level. Ecology Letters 7: 417-426.

17. Huntley B, Berry PM, Cramer W, McDonald AP (1995) Modelling present and potential future ranges of some European higher plants using climate response surfaces. Journal of Biogeography 22: 967-1001.

18. Cleveland WS, Devlin SJ (1998) Locally weighted regresssion: an approach to regression analysis by local fitting. Journal of the American Statistical Association 83: 596-610.

19. Cubasch U, Meehl GA, Boer GJ, Stouffer RJ, Dix M, et al. (2001) Projections of future climate change. In: Johnson CA, editor. Climate Change: The Scientific Basis. Cambridge: Cambridge University Press. pp. 525-582.

20. Nakicenovic N, Swart R, editors (2000) Special Report on Emissions Scenarios. Cambridge, UK: Cambridge University Press.

21. New M, Hulme M, Jones P (1999) Representing twentieth-century space-time climate variability. Part I: Development of a 1961-90 mean monthly terrestrial climatology. Journal of Climate 12: 829-856.

22. Hagemeijer JM, Blair MJ (1997) The EBCC atlas of European breeding birds: Their distribution and abundance. London: T & AD Poyser.

23. Prentice IC, Cramer W, Harrison SP, Leemans R, Monserud RA, et al. (1992) A Global Biome Model Based on Plant Physiology and Dominance, Soil Properties and Climate. Journal of Biogeography 19: 117-134.

24. Hutchinson MF (1989) A new objective method for spatial interpolation of meteorological variables from irregular networks applied to the estimation of monthly mean solar radiation, temperature and windrun. Need for climate and hydrological data in agriculture in southeast Asia: Vol 89/5. Canberra, Australia: CSIRO. pp. 95-104.

25. Metz CE (1978) Basic principles of ROC analysis. Semin Nucl Med 4: 283-298.

26. Araujo MB, Pearson RG, Thuiller W, Erhard M (2005) Validation of species-climate impact models under climate change. Global Change Biology 11: 1504-1513.

27. Huntley B, Green RE, Collingham YC, Willis SG (2007) A climatic atlas of European breeding birds. Barcelona: Lynx Edicions.

28. Huntley B, Collingham YC, Willis SG, Green RE (2008) Potential impacts of climatic change on European breeding birds. PLoS ONE 3: e1439. doi:1410.1371/journal.pone.0001439.

29. Swets JA (1988) Measuring the Accuracy of Diagnostic Systems. Science 240: 1285-1293.

30. Green RE, Collingham YC, Willis SG, Gregory RD, Smith KW, et al. (2008) Performance of climate envelope models in retrodicting recent changes in bird population size from observed climatic change. Biology Letters doi: 10.1098/rsbl.2008.0052.

31. Beerling DJ, Huntley B, Bailey JP (1995) Climate and the distribution of *Fallopia japonica* - use of an introduced species to test the predictive capacity of response surfaces. Journal of Vegetation Science 6: 269-282.

32. Hill JK, Thomas CD, Huntley B (1999) Climate and habitat availability determine 20th century changes in a butterfly's range margin. Proceedings of the Royal Society of London B 266: 1197-1206.

33. Cramp S, Simmons AD, Perrins CM, editors (1977-1994) Handbook of the Birds of Europe, the Middle East and North Africa: the Birds of the Western Palaearctic: Oxford University Press.

34. Burnham KP, Anderson DR (2002) Model selection and multimodel inference: a practical information-theoretic approach. New York, USA.: Springer-Verlag. 488 p.

35. Harvey PH, Pagel MD (1991) The Comparative Method in Evolutionary Biology. Oxford, UK: Oxford University Press.

36. Felsenstein J (1985) Confidence limits on phylogenies: An approach using the bootstrap. Evolution 39: 783-791.

37. Purvis A, Rambaut A (1995) Comparative analysis by independent contrasts (CAIC): an Apple Macintosh application for analysing comparative data. CABIOS 11: 247-251.

38. Sibley CG, Monroe JE (1990) Distribution and taxonomy of birds of the world. New Haven, Conneticut: Yale University Press.

39. data from Worldclim database, available at http://wwwworldclimorg for the period 1960–1990; occasionally 1950–2000 where the former period was not available.

40. Jiguet F, Julliard R, Thomas CD, Dehorter O, Newson SE, et al. (2006) Thermal range predicts bird population resilience to extreme high temperatures. Ecology Letters 9: 1321-1330.

41. Jiguet F, Gadot AS, Julliard R, Newson SE, Couvet D (2007) Climate envelope, life history traits and the resilience of birds facing global change. Global Change Biology 13: 1672-1684.
