# Supplementary material for: An Indicator of the Impact of Climatic Change on European Bird Populations
Source: PLoS One. 2009 Mar 4;4(3):e4678. doi: 10.1371/journal.pone.0004678 (PMC2649536; doi:10.1371/journal.pone.0004678)
Supplement: Text S2 — Supplementary results (0.04 MB DOC) [file pone.0004678.s002.doc]

**Text S2. Supplementary results**

Correlation of population trend with CLIM variables

Scatter diagrams indicated positive relationships between interspecific variation in population trend and that in the CLIM variables derived from climate envelope models (Figure S3).

Effects on population trend of breeding habitat, migratory status and body mass

We fitted multiple regression models in which population trend (LTS) was the dependent variable and CLIM, breeding habitat (HAB), migratory status (MIG) and log body mass (LMS) were independent variables. AICc weights for these models are shown in Table S9. The model that included HAB, MIG and the HAB.MIG interaction had the highest AICc weight for most CLIMs and the second highest for the remaining CLIMs. For some CLIMs the model that included LMS, HAB, MIG and the HAB.MIG interaction had the highest AICc weight, and this model had the second-ranking AICc weight for the other CLIMs. Except for these two models, the other models had low AICc weights for all CLIMs. These results show that there is strong support from the data for effects of HAB and MIG on species’ population trends, and, to a slightly smaller extent, LMS. The univariate models, which included only CLIM with no effect of LMS, HAB or MIG, received very little support from the data. The effect of CLIM was well supported regardless of which other variables were included.

Assessment of whether the regression slope of population trend on CLIMEns varies with breeding habitat, migratory status, body mass or the goodness-of-fit of the climatic envelope model

We considered it possible that the slope of population trend on CLIMEns might differ among classes of breeding habitat and migratory status or with body mass or the goodness-of-fit of the climatic envelope model. The latter was assessed using the area under the curve (AUC) from a Receiver Operating Characteristic plot (see supporting Materials and Methods). We tested the significance of two-way interaction terms comprising CLIMEns and each of LMS, HAB, MIG and AUC in addition to the two regression models, which received predominant support in the comparison of results from 19 regression models described above. None of the interactions approached statistical significance (Table S11).

Assessment of the effect on the population trend vs. CLIM regressions of incomplete coverage in climatic envelope models of the southern range boundary

More than half (55%) of the 108 European bird species used in our regression analysis had part of their breeding range in North Africa, an area not covered by the atlas survey we used to fit the climatic envelope models. Hence, the models might not describe the climatic attributes of the southern range boundary of these species adequately and this might lead to unreliable projections of future range change from the CLIM predictors. We believe that this is not a serious problem because values of the bioclimate variables within the breeding ranges of these species in North Africa are similar to those in southern Europe. However, to test this possibility further we compared the regression of population trend on CLIM for the 59 species that have part of their breeding range in North Africa with that for the 49 species that do not. If our models are rendered unreliable for prediction by inadequacy in describing the southern range boundary, we would expect the regressions for species with part of their range in North Africa to show weaker correlations and smaller regression coefficients in the predicted direction. In fact, the correlations for all but one of the CLIM variables were stronger for species with part of their range in North Africa. A test of the difference in the regression slope of population trends on CLIM between these two classes of species showed no indication of significant differences for any CLIM (Table S12).

Comparison of regressions of population trend on CLIM performed separately for species with positive and negative projected effects of climate change on geographical range

When only species projected to have diminished potential geographical range because of climate change (CLIM-) were considered, both univariate and model averaged multiple regressions of population trend on CLIM variables were all positive and significantly so for five of the six scenarios. CLIMEcB2 was the exception (Table S7). The regression of population trend on CLIMEns was significant for CLIM- species. When only species projected to have increased potential geographical range because of climate change (CLIM+) were considered, univariate and model averaged multiple regressions of population trend on CLIM variables were all positive, but only about half of the relationships were significant (Table S7). The difference in the proportion of scenarios with statistically significant regression is probably due to the smaller sample size for CLIM+ than CLIM- species. The regression of population trend on CLIMEns was significant for CLIM+ species.

The standardized regression coefficient for CLIM+ species was larger than that for CLIM- species for most scenarios, but this difference was only statistically significant for two regressions. For none of the six scenarios was there a significant difference in slope between CLIM- and CLIM+ species for both the univariate and multiple regression. The difference in slope between CLIM- and CLIM+ species for the CLIMEns analysis did not approach statistical significance (Table S7).

Effect of adjusting the climatic impact indicator for the effects of body mass, breeding habitat and migratory status

The adjusted and unadjusted versions of the CII followed similar trajectories (Figure S2) and were highly correlated (*r* = 0.991). For this reason, and for simplicity, we have used the unadjusted version in the main part of the paper.

Comparison of indicator trajectories for indicators based upon CLIM variables derived from different climate change scenarios

The indices for groups of species projected to decrease or increase their potential geographical range because of climate change (CLIM- and CLIM+ species) showed broadly similar patterns of change over time regardless of which of the six climate change scenarios was used to generate CLIM values (Figure S1A and B). This is also the case for the Climatic Impact Indicator, which is the ratio of the indices for these two groups of species (Figure S1C).

Change in climate during the period 1980 – 2002 in bird survey countries

Our intention in using a two-factor (COUNTRY x YEAR) anova model was to allow for the staggered entry of countries into the PECBMS bird survey dataset (see above). Had we not allowed for this and simply calculated the mean climate values for a given year across all countries, we would have been comparing the measured change in bird populations in a subset of countries with the change in climate for all 20 countries. This could mean that changes in temperature might not accurately reflect those in the countries from which the bird survey data were derived. In fact, there is little scope for this to influence our conclusions because the standardized values obtained from the two-factor anova model and those obtained simply by averaging the climate variable across all countries were very similar in most years (Figure S4), and there were very high Pearson correlations between them (*r* = 0.910, 0.986, 0.975 for GDD5, MTCO and MTEMP respectively). Nonetheless, we used the results from the two-factor anova for comparison with the CII.

**Representation of threatened species in the group of species used to calculate the indicator**

The comparison of European threat status (derived from the IUCN Red List Criteria) shows that our dataset provides reasonably good coverage of species in the Vulnerable, Declining, Depleted and Secure categories (Table S8); which together cover 85% of European breeding species. However, there is no coverage of the remaining species in the categories, Critically Endangered, Endangered, Rare and Localised, demonstrating that the rarer and more localised species are unrepresented in our dataset.

**Assessing the representativeness of the species included in the indicator**

We found a near-significant positive correlation between BIE2 log (*T*) and CLIMEns for the indicator species (*r* = 0.137, *P* = 0.068). This is a weaker correlation than that between the 1980 – 2005 population trend from annual PECMBS counts and CLIMEns for the whole of Europe for the 108 indicator species for which we have PECBMS trend estimates (*r* = 0.204, *P* = 0.017). The difference in the strength of the correlation may be caused by the probable lower accuracy of estimates of trend from sources other than annual counting schemes, which are included in the BIE2 scores, and the difference in the time periods covered by the two trend estimates. Indeed, the positive correlation between the PECBMS 1980 – 2005 population trend and the 1990 – 2000 BIE2 log (*T*), whilst highly significant, is not particularly strong (*r* = 0.514, *P* < 0.001).

In contrast to the result for the indicator species, we found no evidence of a positive correlation between BIE2 log (*T*) and CLIMEns for the non-indicator species (*r* = -0.019). Although analysis of covariance did not indicate any significant difference between indicator and non-indicator species in the regression relationships between log (*T*) and CLIMEns (test of main effect of indicator and the two-way interaction indicator x CLIMEns, *F_2,386_* = 0.50, *P* = 0.606), this result suggests that the positive relationship found between log(*T*) and CLIMEns for indicator species cannot be assumed also to apply to non-indicator species. We conducted further analyses to explore this difference in more detail. We found that there was a tendency for the positive relationship found between log (*T*) and CLIMEns to be strongest for the species with the largest breeding populations in Europe as a whole. A regression model for all species in which log (*T*) was regressed on the main effects of CLIMEns and the log of the total European breeding population (in pairs in 2000, from BIE2) and the two-way interaction between log total population and CLIMEns showed that the effect of log population and its interaction with CLIMEns was almost significant (*F_2,386_* = 2.93, *P* = 0.055) and the interaction term itself was highly significant (*F_1,386_* = 5.84, *P* = 0.016). This suggests the possibility that the positive relationship of log (*T*) to CLIMEns extended beyond the indicator species to the more abundant of the non-indicator species. By calculating the correlation of log (*T*) with CLIMEns for subsets of non-indicator species ranked in descending order of total population size, we found that the correlation was statistically significant for the 200 non-indicator species more abundant than 20,000 pairs (*r* = 0.120, *P* = 0.046). This relationship was also significant for the 321 indicator and non-indicator species combined with abundance greater than 20,000 pairs (*r* = 0.116, *P* = 0.019). Hence, our analyses indicate that the positive relationship between recent observed population trend and the projected change in range from climate envelope models extends well beyond the indicator species to the more abundant of European breeding birds in general, which comprise 62% of all species and 82% of species for which the BIE2 data and climate envelope models permitted a test to be made. We speculate that the lack of such a relationship for Europe’s least abundant birds may arise because of greater imperfections in the data on their population trends than for more abundant species, or because our climate envelope models are less reliable in describing causal effects of climate on range boundaries in scarce species. Both explanations are plausible. A previous finding that our climate envelope models of the ranges of European bird species with restricted ranges fit the observed range less well than those of more widely distributed species [1] is consistent with the second explanation.

**Relationships between population trends and independent CRPs based on species’ recent geographical ranges**

As expected, we found significant positive correlations between long-term population trends and TMEAN, TMAX and TMIN, and a significant negative correlation between trends and LAT, when considered alone and when controlling for the potentially confounding effects of species’ body mass, habitat choice and migratory status (Table S4). Population trend also correlated significantly with the additional CRPs after controlling for phylogenetic relationships (Table S5). All of the CRPs were strongly correlated with one another (Table S6). Our analyses suggest that these characteristics of a species’ recent geographical range in Europe were strongly associated with their observed recent trends. This supports our main analyses where we have used independent projections from climate envelope models as the basis for our indicator. We chose to use CLIMEns, rather than latitude or the thermal measures because, unlike them, it provides a clear projection of the direction of change for each species within our study area.

Reference

1. Huntley B, Green RE, Collingham YC, Willis SG (2007) A climatic atlas of European breeding birds. Barcelona: Lynx Edicions.
