# Supplementary material for: An Indicator of the Impact of Climatic Change on European Bird Populations
Source: PLoS One. 2009 Mar 4;4(3):e4678. doi: 10.1371/journal.pone.0004678 (PMC2649536; doi:10.1371/journal.pone.0004678)
Supplement: Text S3 — Synthesis (0.04 MB DOC) [file pone.0004678.s003.doc]

# Text S3. Synthesis

Overall, our analyses indicate that climatic change is having a detectable continent-wide effect at the level of a large species assemblage, including evidence of negative as well as positive effects. Many previous studies of the impacts of climatic change have relied upon changes in the location of range margins [1,2] or latitudinal distribution [3,4], and ours is one of few to document an association between climatic change and change in abundance over a large geographical area. Our work also provides partial support for previous studies that have used bioclimate models with other information to predict potential extinction risk from climate change [5].

While our work has focused on climate envelope modelling, recent efforts have shown the value of integrating land cover changes in bioclimatic modelling, showing how predictions of species distributions can be improved by combining climate and land use information [6-9]. Jetz et al. [10] have gone further in showing how projected climatic and land-use change might impact upon global bird diversity. Future models that combine climatic, land-use and land-cover change might provide the basis for refined climatic impact indicators. However, the uncertainty of model projections of the impact of land cover change and its interactions with climatic change would be even larger than those associated with climatic change alone.

We began this paper by identifying three main barriers to progress in developing indicators of the impacts of climatic change upon biodiversity. The first problem, that of inadequate data, has largely been overcome by combining climatic envelope models fitted to surveys of presence and absence over most of Europe [11] with annual monitoring of the abundance of more than a hundred land birds from 20 countries over a 25-year period [12-13]. The second problem, that of insufficiently validated models, has been addressed by testing climatic envelope model performance in retrodicting long-term trends in abundance of these bird species, rather than just fitting static distributions. The third problem, of uncertainty in climatic change and its consequences, remains very important, but our results show that the pattern of change of the indicators is broadly similar regardless of which climatic change scenario is used (Figure S1). Further work should incorporate uncertainty not just in the nature of future climatic change, but also in species’ responses to that change.

**References**

1. Parmesan C, Yohe G (2003) A globally coherent fingerprint of climate change impacts across natural systems. Nature 421: 37-42.

2. Hickling R, Roy DB, Hill JK, Fox R, Thomas CD (2006) The distributions of a wide range of taxonomic groups are expanding polewards. Global Change Biology 12: 450–455.

3. Grabherr G, Gottfried M, Pauli H (1994) Climate Effects on Mountain Plants. Nature 369: 448-448.

4. La Sorte FA, Thompson III FR (2007) Poleward shifts in winter ranges of North American birds. Ecology 88: 1803-1812.

5. Thomas CD, Cameron A, Green RE, Bakkenes M, Beaumont LJ, et al. (2004) Extinction risk from climate change. Nature 427: 145-148.

6. Luoto M, Heikkinen RK, Poyry J, Saarinen K (2006) Determinants of the biogeographical distribution of butterflies in boreal regions. Journal of Biogeography 33: 1764-1778.

7. Kivinen S, Luoto M, Kuussaari M, Saarinen KM (2007) Effects of land cover and climate on species richness of butterflies in boreal agricultural landscapes. Agriculture Ecosystems and Environment 122: 453-460.

8. Virkkala R, Luoto M, Heikkinen RK, Leikola N (2005) Distribution patterns of boreal marshland birds: modelling the relationships to land cover and climate. Journal of Biogeography 32: 1957-1970.

9. Luoto M, Virkkala R, Heikkinen RK (2007) The role of land cover in bioclimatic models depends on spatial resolution. Global Ecology and Biogeography 16: 34-42.

10. Jetz W, Wilcove DS, Dobson AP (2007) Projected impacts of climate and land-use change on the global diversity of birds. PLoS Biology 5: 1211-1219.

11. Huntley B, Green RE, Collingham YC, Willis SG (2007) A climatic atlas of European breeding birds. Barcelona: Lynx Edicions.

12. Gregory RD, Vorisek P, Van Strien A, Meyling AWG, Jiguet F, et al. (2007) Population trends of widespread woodland birds in Europe. Ibis 149: 78-97.

13. Gregory RD, van Strien A, Vorisek P, Meyling AWG, Noble DG, et al. (2005) Developing indicators for European birds. Philosophical Transactions of the Royal Society B-Biological Sciences 360: 269-288.
