# Supplementary figures and images for: An Indicator of the Impact of Climatic Change on European Bird Populations
Source: PLoS One. 2009 Mar 4;4(3):e4678. doi: 10.1371/journal.pone.0004678 (PMC2649536; doi:10.1371/journal.pone.0004678)

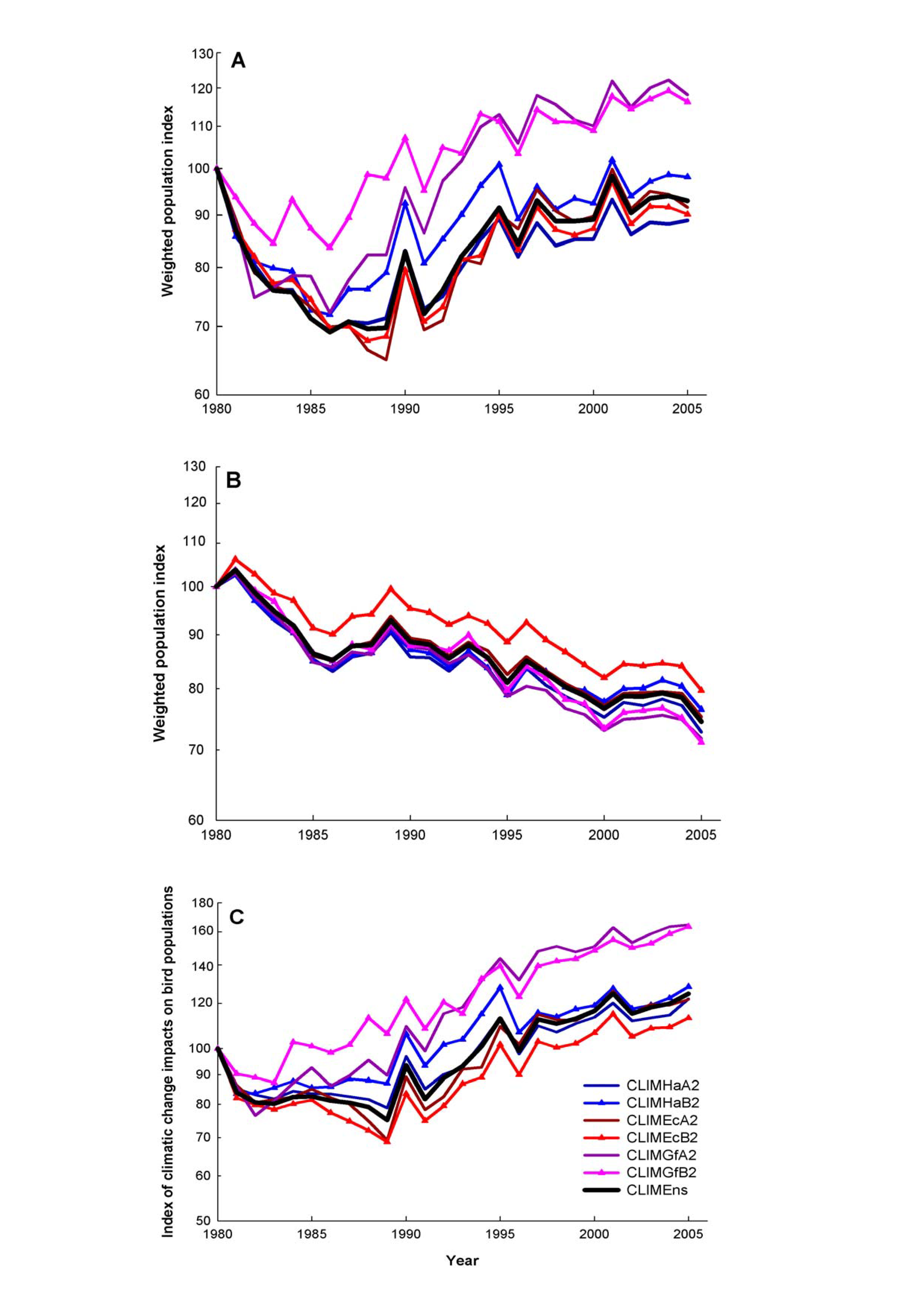

Supplement: Figure S1 — Indicators of the impact of climate change derived from each of the six GCM/emissions scenarios and that based upon their average (CLIMEns). (A) The indicator for bird species predicted to gain potential geographical range under climate change for the 6 scenarios and for their average. (B) The indicator for bird species predicted to lose potential geographical range under climate change for the 6 scenarios and for their average. (C) The Climate Impact Indicator, which is the ratio of the indicator in (A) to that in (B). All indicators are set at 100 in 1980 and are plotted on a logarithmic scale. (6.54 MB TIF) [file pone.0004678.s004.tif]

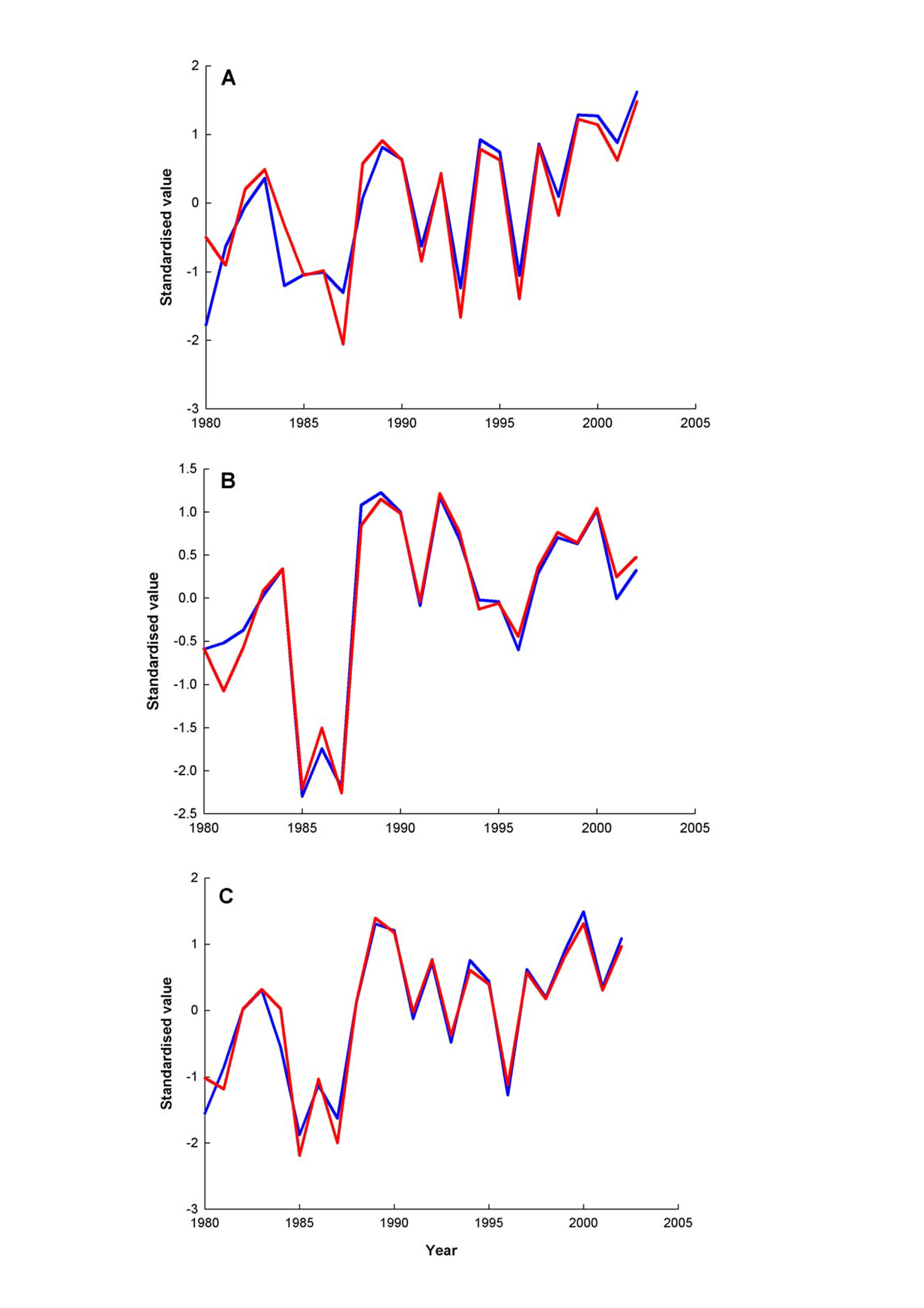

Supplement: Figure S4 — Standardised values of the bioclimate variables. (A) GDD5 - growing days above 5°C, (B) MTCO - the mean temperature of the coldest month, and (C) MTEMP - the mean annual temperature, calculated from the respective means for all 20 countries (blue) and from an anova model taking into account only conditions in the years in which countries contributed bird population survey data (red). (4.93 MB TIF) [file pone.0004678.s007.tif]
