# Supplementary material for: An Indicator of the Impact of Climatic Change on European Bird Populations
Source: PLoS One. 2009 Mar 4;4(3):e4678. doi: 10.1371/journal.pone.0004678 (PMC2649536; doi:10.1371/journal.pone.0004678)
Supplement: Table S1 — Bird species data. (0.26 MB DOC) [file pone.0004678.s008.doc]

Table S1. Bird species data.

| Species | Hab | Mig | Lmass | LTS | SE LTS | LAT | TMEAN | TMAX | TMIN | CLIMHaA2 | CLIMHaB2 | CLIMEcA2 | CLIMEcB2 | CLIMGfA2 | CLIMGfB2 | CLIMEns | Phylogenetic coding |
| --- | --- | --- | --- | --- | --- | --- | --- | --- | --- | --- | --- | --- | --- | --- | --- | --- | --- |
| *Acrocephalus arundinaceus* | IW | LD | 3.303 | 0.031 | 0.016 | 47.780 | 15.787 | 20.864 | 10.853 | 0.157 | 0.238 | -0.088 | -0.005 | 0.034 | 0.171 | 0.084 | BBBBBBBBBABAAAAAAAAAB |
| *Acrocephalus palustris* | O | LD | 2.477 | -0.001 | 0.003 | 51.560 | 14.267 | 19.213 | 8.789 | -0.117 | -0.016 | -0.383 | -0.210 | 0.017 | 0.071 | -0.106 | BBBBBBBBBABAAAAAAAAAABA |
| *Acrocephalus schoenobaenus* | IW | LD | 2.416 | -0.019 | 0.009 | 54.380 | 13.732 | 18.938 | 5.434 | -0.735 | -0.443 | -1.031 | -0.867 | -0.316 | -0.208 | -0.600 | BBBBBBBBBABAAAAAAAAAAA |
| *Acrocephalus scirpaceus* | IW | LD | 2.510 | -0.007 | 0.003 | 49.140 | 14.733 | 20.583 | 9.568 | 0.038 | 0.090 | -0.210 | -0.095 | -0.089 | -0.033 | -0.050 | BBBBBBBBBABAAAAAAAAAABB |
| *Actitis hypoleucos* | IW | LD | 3.945 | -0.021 | 0.005 | 54.230 | 13.579 | 19.894 | 5.190 | -0.276 | -0.111 | -0.498 | -0.325 | -0.145 | -0.125 | -0.247 | BBBBBBABBBB |
| *Aegithalos caudatus* | O | R | 2.104 | 0.007 | 0.004 | 50.630 | 14.478 | 20.298 | 7.238 | -0.122 | -0.047 | -0.241 | -0.116 | -0.072 | -0.037 | -0.106 | BBBBBBBBBABAB |
| *Alauda arvensis* | FA | PM | 3.616 | -0.019 | 0.001 | 52.230 | 14.362 | 20.088 | 6.143 | -0.269 | -0.116 | -0.315 | -0.211 | -0.081 | -0.040 | -0.172 | BBBBBBBBBBAAA |
| *Anthus campestris* | FA | LD | 3.332 | na | na | 45.740 | 16.474 | 20.916 | 11.354 | 0.130 | 0.301 | 0.006 | 0.142 | 0.049 | 0.074 | 0.117 | BBBBBBBBBBBABAAA |
| *Anthus pratensis* | FA | PM | 2.912 | -0.020 | 0.004 | 56.260 | 12.697 | 16.458 | 4.230 | -0.923 | -0.662 | -0.967 | -0.785 | -0.604 | -0.503 | -0.741 | BBBBBBBBBBBABAAC |
| *Anthus trivialis* | FO | LD | 3.223 | -0.030 | 0.001 | 53.920 | 13.764 | 18.565 | 5.365 | -0.448 | -0.258 | -0.595 | -0.428 | -0.137 | -0.052 | -0.320 | BBBBBBBBBBBABAAB |
| *Apus apus* | O | LD | 3.627 | -0.008 | 0.004 | 51.480 | 14.473 | 20.880 | 6.360 | -0.003 | -0.093 | -0.237 | -0.088 | -0.063 | -0.050 | -0.089 | BBBBA |
| *Bonasa bonasia* | FO | R | 6.061 | -0.012 | 0.007 | 57.600 | 12.303 | 16.612 | 5.827 | -0.549 | -0.309 | -0.725 | -0.558 | 0.063 | 0.077 | -0.333 | AB |
| *Calandrella brachydactyla* | FA | LD | 3.135 | na | na | 42.780 | 18.170 | 21.578 | 13.882 | 0.570 | 0.258 | 0.205 | 0.218 | -0.090 | -0.032 | 0.188 | BBBBBBBBBBAC |
| *Carduelis cannabina* | FA | PM | 2.728 | -0.022 | 0.003 | 49.150 | 14.861 | 20.895 | 8.753 | 0.114 | 0.119 | 0.033 | 0.056 | 0.042 | 0.058 | 0.070 | BBBBBBBBBBBBBAAAAAAAA |
| *Carduelis carduelis* | O | PM | 2.747 | 0.017 | 0.003 | 48.770 | 15.074 | 20.951 | 9.543 | 0.195 | 0.089 | 0.038 | 0.088 | 0.062 | 0.066 | 0.090 | BBBBBBBBBBBBBAAAAAABA |
| *Carduelis chloris* | O | PM | 3.325 | 0.003 | 0.002 | 50.730 | 14.612 | 20.752 | 6.623 | -0.005 | -0.002 | -0.052 | -0.031 | -0.007 | 0.008 | -0.015 | BBBBBBBBBBBBBAAAAAB |
| *Carduelis flammea* | O | PM | 2.565 | -0.012 | 0.037 | 60.050 | 10.432 | 14.683 | 4.065 | -1.180 | -0.832 | -1.272 | -0.935 | -0.576 | -0.549 | -0.891 | BBBBBBBBBBBBBAAAAAABB |
| *Carduelis spinus* | FO | PM | 2.674 | -0.010 | 0.004 | 56.120 | 12.703 | 17.214 | 5.360 | -0.789 | -0.600 | -0.872 | -0.607 | -0.413 | -0.330 | -0.602 | BBBBBBBBBBBBBAAAAAAAB |
| *Carpodacus erythrinus* | O | LD | 3.182 | 0.017 | 0.005 | 56.430 | 12.724 | 18.296 | 6.917 | -0.677 | -0.320 | -0.793 | -0.650 | -0.312 | -0.256 | -0.502 | BBBBBBBBBBBBBAAAB |
| *Certhia brachydactyla* | FO | R | 2.140 | -0.006 | 0.007 | 45.730 | 15.313 | 20.926 | 9.569 | -0.233 | -0.028 | -0.263 | -0.167 | -0.026 | 0.038 | -0.113 | BBBBBBBBBAAAAA |
| *Certhia familiaris* | FO | R | 2.197 | 0.000 | 0.003 | 53.860 | 13.429 | 17.821 | 6.396 | -0.656 | -0.416 | -0.813 | -0.646 | -0.211 | -0.132 | -0.479 | BBBBBBBBBAAAAB |
| *Cettia cetti* | IW | R | 2.534 | 0.090 | 0.014 | 41.920 | 17.531 | 21.570 | 11.953 | 0.604 | 0.494 | 0.447 | 0.538 | 0.286 | 0.181 | 0.425 | BBBBBBBBBABAAAAAB |
| *Ciconia ciconia* | FA | LD | 8.153 | 0.026 | 0.014 | 48.560 | 15.273 | 20.887 | 12.130 | -0.294 | -0.073 | -0.532 | -0.498 | 0.078 | 0.176 | -0.190 | BBBBBBAAA |
| *Cisticola juncidis* | IW | R | 2.303 | na | na | 40.590 | 18.391 | 21.838 | 13.187 | 0.643 | 0.519 | 0.535 | 0.595 | 0.319 | 0.195 | 0.468 | BBBBBBBBBABAAAAB |
| *Coccothraustes coccothraustes* | FO | PM | 3.989 | 0.021 | 0.007 | 49.290 | 14.837 | 20.312 | 9.344 | -0.123 | -0.132 | -0.232 | -0.724 | -0.035 | 0.039 | -0.201 | BBBBBBBBBBBBBAB |
| *Columba oenas* | FO | PM | 5.635 | 0.008 | 0.004 | 51.210 | 14.098 | 19.842 | 9.025 | -0.512 | -0.318 | -0.770 | -0.652 | -0.318 | -0.160 | -0.455 | BBBBBAAB |
| *Columba palumbus* | O | PM | 6.194 | 0.019 | 0.002 | 51.660 | 14.312 | 20.382 | 6.305 | -0.163 | -0.185 | -0.279 | -0.150 | -0.090 | -0.046 | -0.152 | BBBBBAAA |
| *Corvus corax* | O | R | 7.054 | 0.046 | 0.005 | 52.970 | 14.009 | 20.906 | 4.636 | -0.218 | -0.081 | -0.490 | -0.327 | -0.099 | -0.083 | -0.216 | BBBBBBBABAAAAA |
| *Corvus corone* | O | PM | 6.346 | 0.006 | 0.002 | 52.400 | 14.358 | 20.527 | 5.443 | -0.062 | -0.099 | -0.217 | -0.138 | -0.111 | -0.083 | -0.118 | BBBBBBBABAAAAB |
| *Corvus frugilegus* | FA | PM | 6.190 | 0.015 | 0.004 | 50.790 | 14.662 | 18.916 | 10.173 | -0.590 | -0.398 | -0.933 | -0.819 | -0.212 | -0.047 | -0.500 | BBBBBBBABAAABA |
| *Corvus monedula* | O | PM | 5.505 | -0.007 | 0.004 | 50.320 | 14.748 | 20.653 | 8.528 | -0.123 | -0.020 | -0.466 | -0.262 | -0.255 | -0.128 | -0.209 | BBBBBBBABAAABB |
| *Cuculus canorus* | O | LD | 4.727 | -0.013 | 0.002 | 52.070 | 14.352 | 20.520 | 5.642 | -0.057 | -0.123 | -0.123 | -0.093 | -0.054 | -0.042 | -0.082 | BBBA |
| *Delichon urbica* | O | LD | 2.674 | -0.020 | 0.006 | 51.410 | 14.512 | 20.806 | 5.781 | -0.023 | -0.019 | -0.160 | -0.101 | -0.050 | -0.038 | -0.065 | BBBBBBBBBABAABA |
| *Dendrocopos major* | O | R | 4.402 | 0.011 | 0.002 | 52.410 | 14.356 | 19.789 | 6.235 | -0.240 | -0.135 | -0.295 | -0.222 | -0.082 | -0.021 | -0.166 | BAAAAA |
| *Dendrocopos minor* | FO | R | 2.986 | -0.076 | 0.026 | 53.660 | 14.042 | 18.995 | 5.556 | -0.451 | -0.146 | -0.627 | -0.442 | -0.127 | -0.046 | -0.307 | BAAAAB |
| *Dryocopus martius* | FO | R | 5.771 | 0.021 | 0.005 | 54.470 | 13.596 | 18.205 | 6.254 | -0.629 | -0.406 | -0.807 | -0.642 | -0.160 | -0.094 | -0.457 | BAAB |
| *Emberiza cia* | O | PM | 3.219 | na | na | 41.690 | 16.524 | 21.563 | 7.548 | 0.463 | 0.297 | 0.328 | 0.391 | 0.398 | 0.391 | 0.378 | BBBBBBBBBBBBAAAC |
| *Emberiza cirlus* | FA | R | 3.140 | 0.033 | 0.015 | 42.710 | 16.851 | 21.365 | 9.609 | 0.463 | 0.411 | 0.521 | 0.538 | 0.346 | 0.327 | 0.434 | BBBBBBBBBBBBAAAA |
| *Emberiza citrinella* | FA | PM | 3.277 | -0.021 | 0.001 | 53.520 | 13.857 | 18.545 | 5.948 | -0.366 | -0.230 | -0.472 | -0.329 | -0.125 | -0.049 | -0.262 | BBBBBBBBBBBBAAAB |
| *Emberiza hortulana* | FA | LD | 3.170 | -0.014 | 0.009 | 50.760 | 14.955 | 20.728 | 7.588 | 0.245 | 0.227 | 0.073 | 0.174 | 0.007 | 0.106 | 0.139 | BBBBBBBBBBBBAAAD |
| *Emberiza schoeniclus* | IW | PM | 2.907 | -0.004 | 0.003 | 54.500 | 13.645 | 19.280 | 5.218 | -0.554 | -0.319 | -0.647 | -0.479 | -0.155 | -0.109 | -0.377 | BBBBBBBBBBBBAAB |
| *Erithacus rubecula* | O | PM | 2.901 | 0.012 | 0.001 | 52.360 | 14.240 | 20.158 | 5.822 | -0.266 | -0.174 | -0.370 | -0.246 | -0.121 | -0.091 | -0.211 | BBBBBBBBAAAB |
| *Falco tinnunculus* | FA | PM | 5.380 | -0.008 | 0.004 | 51.380 | 14.579 | 20.850 | 5.956 | -0.041 | -0.079 | -0.190 | -0.099 | -0.051 | -0.023 | -0.080 | BBBBBBAABB |
| *Ficedula albicollis* | FO | LD | 2.332 | 0.035 | 0.007 | 49.110 | 15.009 | 18.358 | 10.843 | -0.071 | 0.163 | -0.718 | -0.770 | 0.304 | 0.274 | -0.136 | BBBBBBBBAAAABBAA |
| *Ficedula hypoleuca* | FO | LD | 2.451 | -0.012 | 0.002 | 55.770 | 13.174 | 18.013 | 5.114 | -0.948 | -0.724 | -0.976 | -0.805 | -0.643 | -0.471 | -0.761 | BBBBBBBBAAAABBAB |
| *Fringilla coelebs* | O | PM | 3.040 | 0.000 | 0.001 | 51.750 | 14.471 | 20.740 | 5.917 | -0.038 | -0.072 | -0.117 | -0.073 | -0.052 | -0.046 | -0.066 | BBBBBBBBBBBBBBA |
| *Fringilla montifringilla* | O | PM | 3.178 | -0.034 | 0.009 | 62.090 | 9.425 | 14.084 | 4.103 | -1.080 | -0.573 | -0.916 | -0.621 | -0.315 | -0.282 | -0.631 | BBBBBBBBBBBBBBB |
| *Galerida cristata* | FA | R | 3.735 | -0.147 | 0.051 | 46.060 | 16.359 | 21.210 | 12.266 | 0.234 | 0.096 | 0.082 | 0.138 | 0.014 | 0.038 | 0.101 | BBBBBBBBBBAABA |
| *Galerida theklae* | FA | R | 3.597 | na | na | 39.760 | 18.131 | 21.709 | 12.625 | -0.617 | -0.849 | -0.547 | -0.481 | -0.134 | 0.020 | -0.435 | BBBBBBBBBBAABB |
| *Gallinago gallinago* | O | PM | 4.754 | -0.023 | 0.003 | 56.260 | 12.901 | 17.550 | 4.364 | -0.952 | -0.711 | -1.038 | -0.868 | -0.595 | -0.457 | -0.770 | BBBBBBABBBA |
| *Garrulus glandarius* | FO | PM | 5.081 | 0.001 | 0.003 | 50.930 | 14.541 | 20.451 | 6.620 | -0.024 | -0.075 | -0.174 | -0.086 | -0.052 | -0.015 | -0.071 | BBBBBBBABABB |
| *Hippolais icterina* | O | LD | 2.681 | -0.019 | 0.003 | 53.800 | 13.671 | 18.191 | 6.022 | -0.697 | -0.430 | -0.799 | -0.655 | -0.391 | -0.288 | -0.543 | BBBBBBBBBABAAAAAAAABA |
| *Hippolais polyglotta* | O | LD | 2.398 | -0.019 | 0.009 | 43.210 | 16.248 | 21.311 | 9.654 | 0.206 | 0.194 | 0.183 | 0.286 | 0.182 | 0.193 | 0.207 | BBBBBBBBBABAAAAAAAABB |
| *Hirundo rupestris* | O | PM | 3.157 | na | na | 40.830 | 16.823 | 21.608 | 7.124 | 0.583 | 0.389 | 0.366 | 0.418 | 0.149 | 0.217 | 0.354 | BBBBBBBBBABAABBB |
| *Hirundo rustica* | FA | LD | 2.760 | -0.006 | 0.002 | 51.400 | 14.524 | 20.764 | 6.001 | 0.001 | -0.055 | -0.080 | -0.058 | -0.029 | 0.000 | -0.037 | BBBBBBBBBABAABBA |
| *Jynx torquilla* | O | LD | 3.512 | -0.049 | 0.007 | 52.830 | 14.392 | 19.683 | 5.895 | -0.244 | -0.114 | -0.426 | -0.260 | -0.130 | -0.054 | -0.205 | BAB |
| *Lanius collurio* | FA | LD | 3.398 | 0.001 | 0.006 | 51.060 | 14.734 | 19.903 | 7.845 | 0.063 | 0.136 | -0.145 | -0.008 | 0.044 | 0.063 | 0.026 | BBBBBBBAAA |
| *Lanius senator* | FA | LD | 3.555 | na | na | 41.340 | 17.532 | 21.756 | 12.243 | 0.606 | 0.528 | 0.427 | 0.471 | 0.162 | 0.221 | 0.402 | BBBBBBBAAB |
| *Limosa limosa* | FA | LD | 5.728 | na | na | 52.320 | 14.458 | 18.301 | 4.797 | -0.537 | -0.461 | -0.888 | -0.996 | -0.131 | 0.023 | -0.498 | BBBBBBABBA |
| *Locustella fluviatilis* | O | LD | 2.896 | -0.011 | 0.007 | 52.840 | 14.456 | 18.107 | 10.484 | -0.310 | -0.054 | -0.673 | -0.581 | 0.054 | 0.067 | -0.249 | BBBBBBBBBABAAAACA |
| *Locustella naevia* | O | LD | 2.588 | -0.013 | 0.007 | 52.810 | 13.628 | 17.628 | 9.129 | -0.607 | -0.484 | -0.729 | -0.636 | -0.304 | -0.199 | -0.493 | BBBBBBBBBABAAAACB |
| *Lullula arborea* | O | PM | 3.292 | 0.043 | 0.032 | 47.750 | 15.049 | 21.012 | 10.189 | 0.000 | -0.031 | -0.100 | -0.034 | -0.141 | -0.042 | -0.058 | BBBBBBBBBBAB |
| *Luscinia luscinia* | O | LD | 3.170 | -0.022 | 0.003 | 53.680 | 14.210 | 18.726 | 9.334 | -0.464 | -0.207 | -0.706 | -0.515 | -0.297 | -0.253 | -0.407 | BBBBBBBBAAAAAAAA |
| *Luscinia megarhynchos* | O | LD | 2.907 | -0.038 | 0.006 | 44.930 | 16.003 | 21.293 | 10.578 | 0.235 | 0.155 | 0.084 | 0.107 | 0.043 | 0.098 | 0.120 | BBBBBBBBAAAAAAAB |
| *Melanocorypha calandra* | FA | R | 4.082 | na | na | 43.080 | 18.135 | 21.711 | 14.293 | 0.394 | -0.125 | 0.281 | 0.136 | -0.125 | -0.093 | 0.078 | BBBBBBBBBBAD |
| *Merops apiaster* | O | LD | 4.036 | 0.035 | 0.036 | 44.240 | 17.538 | 21.379 | 12.825 | 0.746 | 0.652 | 0.332 | 0.371 | 0.465 | 0.451 | 0.503 | BBABB |
| *Miliaria calandra* | FA | PM | 4.047 | -0.035 | 0.006 | 46.000 | 15.674 | 21.192 | 10.674 | 0.197 | 0.156 | 0.031 | 0.093 | 0.001 | 0.059 | 0.089 | BBBBBBBBBBBBAB |
| *Motacilla alba* | O | PM | 3.045 | -0.004 | 0.002 | 52.690 | 14.256 | 20.612 | 4.826 | -0.103 | -0.108 | -0.196 | -0.151 | -0.108 | -0.068 | -0.122 | BBBBBBBBBBBABABA |
| *Motacilla cinerea* | IW | PM | 2.845 | -0.025 | 0.006 | 48.410 | 14.518 | 20.560 | 6.724 | -0.065 | -0.077 | -0.247 | -0.126 | -0.044 | -0.010 | -0.095 | BBBBBBBBBBBABABBA |
| *Motacilla flava* | FA | LD | 2.632 | -0.013 | 0.005 | 52.340 | 14.520 | 20.438 | 5.525 | -0.216 | -0.101 | -0.322 | -0.255 | -0.063 | 0.002 | -0.159 | BBBBBBBBBBBABABBB |
| *Muscicapa striata* | O | LD | 2.681 | -0.031 | 0.005 | 52.030 | 14.323 | 20.732 | 5.730 | -0.092 | -0.051 | -0.407 | -0.269 | -0.114 | -0.081 | -0.169 | BBBBBBBBAAAABBB |
| *Nucifraga caryocatactes* | FO | R | 5.130 | -0.030 | 0.015 | 54.910 | 13.058 | 16.348 | 6.597 | -0.347 | -0.263 | -0.461 | -0.461 | -0.361 | -0.326 | -0.370 | BBBBBBBABAABA |
| *Oenanthe hispanica* | FA | LD | 2.848 | na | na | 40.230 | 18.335 | 22.008 | 12.685 | 0.504 | 0.315 | 0.237 | 0.293 | -0.044 | -0.031 | 0.212 | BBBBBBBBAAAAABA |
| *Oenanthe oenanthe* | O | LD | 3.105 | -0.054 | 0.014 | 53.220 | 13.951 | 20.476 | 4.666 | -0.361 | -0.208 | -0.556 | -0.373 | -0.339 | -0.184 | -0.337 | BBBBBBBBAAAAABB |
| *Oriolus oriolus* | O | LD | 4.369 | 0.017 | 0.005 | 49.240 | 15.273 | 20.589 | 10.081 | 0.212 | 0.105 | 0.124 | 0.146 | 0.129 | 0.153 | 0.145 | BBBBBBBABB |
| *Parus ater* | FO | PM | 2.208 | -0.002 | 0.003 | 52.220 | 13.794 | 20.173 | 6.649 | -0.279 | -0.213 | -0.368 | -0.262 | -0.186 | -0.112 | -0.237 | BBBBBBBBBABBBA |
| *Parus caeruleus* | O | PM | 2.588 | 0.009 | 0.001 | 50.150 | 14.667 | 20.597 | 7.160 | 0.029 | -0.007 | -0.027 | -0.016 | 0.010 | 0.011 | 0.000 | BBBBBBBBBABBAA |
| *Parus cristatus* | FO | R | 2.322 | -0.019 | 0.006 | 52.120 | 13.893 | 20.192 | 6.634 | -0.549 | -0.468 | -0.525 | -0.387 | -0.204 | -0.109 | -0.373 | BBBBBBBBBABBBBA |
| *Parus major* | O | PM | 2.944 | -0.001 | 0.001 | 51.840 | 14.499 | 20.799 | 5.813 | -0.010 | -0.085 | -0.088 | -0.058 | -0.029 | -0.010 | -0.047 | BBBBBBBBBABBAB |
| *Parus montanus* | FO | R | 2.322 | -0.038 | 0.004 | 55.870 | 13.122 | 16.870 | 4.992 | -0.787 | -0.602 | -0.806 | -0.657 | -0.310 | -0.145 | -0.551 | BBBBBBBBBABBBBBA |
| *Parus palustris* | FO | R | 2.361 | -0.026 | 0.004 | 50.080 | 14.328 | 18.614 | 7.093 | -0.031 | 0.022 | -0.354 | -0.155 | -0.037 | 0.030 | -0.087 | BBBBBBBBBABBBBBB |
| *Passer domesticus* | O | R | 3.311 | -0.018 | 0.002 | 52.450 | 14.343 | 20.848 | 5.673 | -0.259 | -0.250 | -0.234 | -0.202 | -0.126 | -0.049 | -0.187 | BBBBBBBBBBBAABA |
| *Passer montanus* | FA | R | 3.091 | -0.019 | 0.004 | 49.920 | 14.906 | 20.549 | 8.465 | -0.023 | 0.018 | -0.167 | -0.050 | -0.069 | 0.005 | -0.048 | BBBBBBBBBBBAABB |
| *Perdix perdix* | FA | R | 5.943 | -0.076 | 0.013 | 49.600 | 14.674 | 19.135 | 9.878 | -0.087 | 0.060 | -0.309 | -0.163 | -0.073 | 0.034 | -0.090 | AA |
| *Petronia petronia* | FA | R | 3.418 | na | na | 40.550 | 17.656 | 21.686 | 10.572 | -0.584 | -0.610 | -0.560 | -0.397 | -0.483 | -0.312 | -0.491 | BBBBBBBBBBBAAA |
| *Phoenicurus ochruros* | O | PM | 2.803 | 0.004 | 0.004 | 47.740 | 14.983 | 20.384 | 9.405 | -0.050 | -0.057 | -0.146 | -0.083 | 0.027 | 0.068 | -0.040 | BBBBBBBBAAAAAABA |
| *Phoenicurus phoenicurus* | FO | LD | 2.674 | -0.002 | 0.003 | 53.880 | 13.724 | 19.243 | 5.426 | -0.361 | -0.184 | -0.762 | -0.454 | -0.204 | -0.119 | -0.348 | BBBBBBBBAAAAAABB |
| *Phylloscopus bonelli* | FO | LD | 2.186 | -0.034 | 0.023 | 43.850 | 15.790 | 21.326 | 7.229 | 0.072 | 0.029 | -0.252 | -0.049 | -0.168 | 0.102 | -0.045 | BBBBBBBBBABAAAAAABA |
| *Phylloscopus collybita* | FO | LD | 2.015 | 0.028 | 0.001 | 52.690 | 14.187 | 19.432 | 6.216 | -0.206 | -0.103 | -0.280 | -0.192 | -0.074 | -0.026 | -0.147 | BBBBBBBBBABAAAAAABBA |
| *Phylloscopus sibilatrix* | FO | LD | 2.104 | -0.028 | 0.003 | 53.670 | 13.695 | 18.190 | 6.367 | -0.471 | -0.287 | -0.720 | -0.501 | -0.224 | -0.124 | -0.388 | BBBBBBBBBABAAAAAABC |
| *Phylloscopus trochilus* | O | LD | 2.163 | -0.018 | 0.001 | 55.740 | 13.119 | 17.277 | 5.003 | -0.856 | -0.633 | -0.926 | -0.787 | -0.518 | -0.342 | -0.677 | BBBBBBBBBABAAAAAABBB |
| *Pica pica* | O | R | 5.112 | -0.005 | 0.002 | 52.310 | 14.424 | 20.494 | 5.577 | -0.193 | -0.129 | -0.377 | -0.196 | -0.106 | -0.039 | -0.174 | BBBBBBBABABA |
| *Picus canus* | FO | R | 4.920 | 0.014 | 0.024 | 51.450 | 14.403 | 18.445 | 6.700 | -0.243 | -0.036 | -0.384 | -0.361 | 0.116 | 0.243 | -0.111 | BAAABA |
| *Picus viridis* | O | R | 5.170 | 0.021 | 0.004 | 48.860 | 14.691 | 20.142 | 7.350 | -0.071 | -0.025 | -0.319 | -0.110 | -0.056 | 0.009 | -0.095 | BAAABB |
| *Prunella modularis* | O | PM | 2.981 | -0.014 | 0.001 | 54.440 | 13.383 | 18.284 | 5.285 | -0.540 | -0.409 | -0.646 | -0.450 | -0.193 | -0.122 | -0.393 | BBBBBBBBBBBABB |
| *Pyrrhocorax pyrrhocorax* | O | R | 5.720 | na | na | 41.620 | 16.078 | 21.604 | 8.063 | -0.491 | -0.577 | -0.958 | -0.405 | -0.412 | -0.365 | -0.535 | BBBBBBBABAABB |
| *Pyrrhula pyrrhula* | FO | PM | 3.082 | -0.014 | 0.003 | 54.880 | 13.277 | 17.812 | 5.472 | -0.513 | -0.355 | -0.673 | -0.440 | -0.194 | -0.115 | -0.382 | BBBBBBBBBBBBBAAB |
| *Regulus ignicapilla* | FO | PM | 1.723 | 0.006 | 0.009 | 46.690 | 14.772 | 20.149 | 8.818 | -0.252 | -0.392 | -0.476 | -0.333 | -0.240 | -0.125 | -0.303 | BBBBBBBBBABAAABA |
| *Regulus regulus* | FO | PM | 1.740 | -0.005 | 0.002 | 54.920 | 13.202 | 17.926 | 5.981 | -0.615 | -0.454 | -0.772 | -0.586 | -0.312 | -0.248 | -0.498 | BBBBBBBBBABAAABB |
| *Saxicola rubetra* | FA | LD | 2.809 | -0.018 | 0.006 | 54.150 | 13.708 | 18.837 | 5.609 | -0.721 | -0.366 | -0.791 | -0.664 | -0.320 | -0.223 | -0.514 | BBBBBBBBAAAABAA |
| *Saxicola torquata* | FA | PM | 2.728 | -0.006 | 0.060 | 47.800 | 15.689 | 21.161 | 9.075 | 0.213 | 0.148 | 0.078 | 0.120 | 0.198 | 0.184 | 0.157 | BBBBBBBBAAAABAB |
| *Serinus serinus* | FA | PM | 2.416 | -0.036 | 0.005 | 46.010 | 15.458 | 21.122 | 9.969 | 0.281 | 0.210 | 0.174 | 0.166 | 0.139 | 0.142 | 0.186 | BBBBBBBBBBBBBAAAAB |
| *Sitta europaea* | FO | R | 3.091 | 0.011 | 0.003 | 50.440 | 14.734 | 19.970 | 7.802 | -0.025 | 0.012 | -0.216 | -0.073 | 0.001 | 0.029 | -0.045 | BBBBBBBBBAAB |
| *Streptopelia decaocto* | O | R | 4.984 | 0.020 | 0.003 | 49.710 | 14.733 | 20.402 | 7.869 | 0.161 | 0.158 | -0.040 | 0.040 | 0.034 | 0.019 | 0.062 | BBBBBABA |
| *Streptopelia turtur* | FA | LD | 4.883 | -0.037 | 0.004 | 48.410 | 15.419 | 21.108 | 10.101 | 0.341 | 0.263 | 0.316 | 0.281 | 0.239 | 0.242 | 0.280 | BBBBBABB |
| *Sturnus unicolor* | FA | R | 4.508 | na | na | 39.860 | 17.882 | 21.783 | 12.76 | -0.685 | -0.611 | -0.099 | -0.285 | -0.099 | -0.030 | -0.301 | BBBBBBBBABB |
| *Sturnus vulgaris* | FA | PM | 4.381 | -0.025 | 0.004 | 53.060 | 14.060 | 19.377 | 5.786 | -0.197 | -0.077 | -0.509 | -0.255 | -0.121 | -0.066 | -0.204 | BBBBBBBBABA |
| *Sylvia atricapilla* | O | PM | 2.741 | 0.026 | 0.001 | 50.620 | 14.605 | 20.483 | 6.659 | -0.058 | -0.031 | -0.073 | -0.013 | 0.008 | 0.015 | -0.025 | BBBBBBBBBABAAAAAAABAA |
| *Sylvia borin* | O | LD | 2.632 | -0.008 | 0.001 | 53.910 | 13.738 | 19.196 | 5.459 | -0.559 | -0.426 | -0.795 | -0.551 | -0.207 | -0.079 | -0.436 | BBBBBBBBBABAAAAAAABAB |
| *Sylvia cantillans* | O | LD | 2.380 | 0.006 | 0.022 | 40.400 | 18.355 | 21.897 | 12.191 | 0.822 | 0.680 | 0.703 | 0.688 | 0.195 | 0.072 | 0.527 | BBBBBBBBBABAAAAAAABBBBBA |
| *Sylvia communis* | FA | LD | 2.674 | 0.010 | 0.001 | 51.190 | 14.586 | 20.368 | 7.634 | 0.036 | 0.030 | -0.217 | -0.066 | -0.011 | -0.005 | -0.039 | BBBBBBBBBABAAAAAAABBBA |
| *Sylvia curruca* | O | LD | 2.313 | 0.000 | 0.002 | 53.490 | 13.845 | 19.102 | 6.455 | -0.600 | -0.326 | -0.703 | -0.543 | -0.108 | -0.037 | -0.386 | BBBBBBBBBABAAAAAAABBA |
| *Sylvia melanocephala* | O | PM | 2.425 | 0.027 | 0.014 | 40.200 | 18.741 | 21.982 | 12.728 | 0.878 | 0.804 | 0.988 | 0.801 | 0.323 | 0.168 | 0.660 | BBBBBBBBBABAAAAAAABBBBBB |
| *Sylvia undata* | O | PM | 2.251 | na | na | 41.880 | 16.920 | 21.453 | 12.454 | -0.147 | -0.125 | 0.192 | 0.118 | 0.016 | 0.133 | 0.031 | BBBBBBBBBABAAAAAAABBBBA |
| *Troglodytes troglodytes* | O | PM | 2.186 | 0.018 | 0.001 | 50.950 | 14.204 | 20.605 | 5.676 | -0.076 | -0.096 | -0.141 | -0.101 | -0.045 | -0.031 | -0.082 | BBBBBBBBBAAAB |
| *Turdus iliacus* | O | PM | 4.114 | 0.000 | 0.002 | 59.790 | 10.800 | 16.157 | 3.906 | -0.902 | -0.466 | -0.936 | -0.685 | -0.405 | -0.292 | -0.614 | BBBBBBBBAABBC |
| *Turdus merula* | O | PM | 4.727 | 0.007 | 0.001 | 50.190 | 14.586 | 20.748 | 6.293 | 0.036 | -0.040 | -0.015 | 0.013 | -0.005 | -0.003 | -0.002 | BBBBBBBBAABA |
| *Turdus philomelos* | O | PM | 4.199 | -0.003 | 0.001 | 53.820 | 13.756 | 18.823 | 5.470 | -0.317 | -0.235 | -0.473 | -0.304 | -0.098 | -0.041 | -0.245 | BBBBBBBBAABBA |
| *Turdus pilaris* | O | PM | 4.644 | 0.008 | 0.002 | 56.390 | 13.059 | 17.169 | 4.770 | -0.805 | -0.642 | -0.763 | -0.638 | -0.359 | -0.234 | -0.573 | BBBBBBBBAABBD |
| *Turdus viscivorus* | FO | PM | 4.745 | -0.012 | 0.003 | 52.270 | 13.938 | 20.164 | 6.571 | -0.385 | -0.253 | -0.540 | -0.360 | -0.104 | -0.060 | -0.284 | BBBBBBBBAABBB |
| *Upupa epops* | FA | LD | 4.117 | 0.012 | 0.044 | 46.140 | 16.156 | 21.107 | 10.292 | 0.433 | 0.311 | 0.240 | 0.290 | 0.291 | 0.311 | 0.313 | BBAA |
| *Vanellus vanellus* | FA | PM | 5.421 | -0.041 | 0.004 | 54.320 | 13.771 | 19.460 | 5.588 | -0.535 | -0.377 | -0.853 | -0.613 | -0.324 | -0.182 | -0.481 | BBBBBBABAA |

Columns describe species’ habitat choice (Hab: IN=inland wetland, FA=farmland, FO=forest, O=other), migratory behaviour (Mig: LD=long-distance migrant, PM=Partial migrant, R=resident), body mass (Lmass), long-term population trend (LTS), error associated with the trend (SE LTS), Latitude (LAT), TMEAN, TMAX, TMIN, CLIMHaA2, CLIMHaB2, CLIMEcA2, CLIMEcB2, CLIMGfA2, CLIMGfB2, CLIMEns, and the coding used in the phylogenetic analyses (see Methods)
