# Supplementary material for: An Indicator of the Impact of Climatic Change on European Bird Populations
Source: PLoS One. 2009 Mar 4;4(3):e4678. doi: 10.1371/journal.pone.0004678 (PMC2649536; doi:10.1371/journal.pone.0004678)
Supplement: Table S2 — Countries providing data for the PECBMS scheme and the period of monitoring used for analysis in each country. (0.02 MB DOC) [file pone.0004678.s009.doc]

Table S2. Countries providing data for the PECBMS scheme and the period of monitoring used for analysis in each country.

| **Country** | **Monitoring Period** |
| --- | --- |
| Austria | 1998 – 2005 |
| Belgium | 1990 – 2005 |
| Czech Republic | 1982 – 2005 |
| Denmark | 1980 – 2005 |
| Estonia | 1983 – 2000 |
| Finland | 1983 – 2005 |
| France | 1989 – 2005 |
| Germany | 1989 – 2005 |
| Hungary | 1999 – 2005 |
| Ireland | 1998 – 2005 |
| Italy | 2000 – 2005 |
| Latvia | 1995 – 2005 |
| Netherlands | 1990 – 2005 |
| Norway | 1995 – 2005 |
| Poland | 2000 – 2005 |
| Portugal | 2004 – 2005 |
| Spain | 1996 – 2005 |
| Sweden | 1980 – 2005 |
| Switzerland | 1999 – 2005 |
| United Kingdom | 1980 – 2005 |
