# Supplementary material for: An Indicator of the Impact of Climatic Change on European Bird Populations
Source: PLoS One. 2009 Mar 4;4(3):e4678. doi: 10.1371/journal.pone.0004678 (PMC2649536; doi:10.1371/journal.pone.0004678)
Supplement: Table S3 — Expected mechanism and direction of effects of Climate Response Predictors (CRPs) on long-term trends in European breeding bird populations. (0.02 MB DOC) [file pone.0004678.s010.doc]

Table S3. Expected mechanism and direction of effects of Climate Response Predictors (CRPs) on long-term trends in European breeding bird populations.

| **CRP** | **Expected direction of effect** | **Justification** |
| --- | --- | --- |
| CLIMHaA2  CLIMHaB2  CLIMEcA2  CLIMEcB2  CLIMGfA2  CLIMGfB2  CLIMEns  CST | + | Populations of species whose potential geographical range is projected to expand (or contract) because of climate change are expected to increase (or decrease) because of the well-established positive relationship between abundance and geographical range size (*38*). |
| Latitude | - | Populations located at middle latitudes of species whose current breeding range lies predominantly at higher (lower) latitude are expected to decrease (increase) because warming will render the climate less (more) favourable. |
| TMEAN | + | Populations of species with a high (low) recent average temperature in their current breeding range are expected to increase (decrease) because warming will render the climate more (less) favourable. |
| TMAX | + | Populations of species whose current breeding range extends to areas with relatively high temperatures are expected to increase because warming will render the local climate more favourable. |
| TMIN | + | Populations of species whose current breeding range does not extend into areas with relatively low temperatures are expected to increase because warming will render the local climate more favourable. |
