# Supplementary material for: An Indicator of the Impact of Climatic Change on European Bird Populations
Source: PLoS One. 2009 Mar 4;4(3):e4678. doi: 10.1371/journal.pone.0004678 (PMC2649536; doi:10.1371/journal.pone.0004678)
Supplement: Table S4 — Results of OLS regression of long-term population trend on CRPs. (0.04 MB DOC) [file pone.0004678.s011.doc]

Table S4. Results of OLS regression of long-term population trend on CRPs.

| **CRP variable** | **beta** | **SE** | ***t*** | ***P* 1-tailed** | **Model averaging** |
| --- | --- | --- | --- | --- | --- |
| CLIMEcA2 | 0.208 | 0.095 | 2.18 | 0.01446 | no |
|  | 0.225 | 0.091 | 2.48 | 0.00666 | yes |
| CLIMEcB2 | 0.169 | 0.096 | 1.76 | 0.03918 | no |
|  | 0.180 | 0.093 | 1.93 | 0.02688 | yes |
| CLIMGfA2 | 0.301 | 0.093 | 3.25 | 0.00057 | no |
|  | 0.342 | 0.083 | 4.11 | 0.00002 | yes |
| CLIMGfB2 | 0.253 | 0.094 | 2.69 | 0.00353 | no |
|  | 0.331 | 0.084 | 3.94 | 0.00004 | yes |
| CLIMHaA2 | 0.219 | 0.095 | 2.31 | 0.01053 | no |
|  | 0.238 | 0.088 | 2.70 | 0.00348 | yes |
| CLIMHaB2 | 0.230 | 0.095 | 2.43 | 0.00748 | no |
|  | 0.273 | 0.089 | 3.08 | 0.00104 | yes |
| CLIMEns | 0.231 | 0.095 | 2.44 | 0.00727 | no |
|  | 0.262 | 0.088 | 2.96 | 0.00154 | yes |
| CST | 0.114 | 0.097 | 1.18 | 0.11938 | no |
|  | 0.149 | 0.091 | 1.64 | 0.05029 | yes |
| LAT | -0.206 | 0.095 | -2.17 | 0.01502 | no |
|  | -0.259 | 0.088 | -2.95 | 0.00158 | yes |
| TMEAN | 0.211 | 0.095 | 2.22 | 0.01328 | no |
|  | 0.272 | 0.088 | 3.07 | 0.00106 | yes |
| TMAX | 0.183 | 0.096 | 1.92 | 0.02770 | no |
|  | 0.181 | 0.090 | 2.01 | 0.02206 | yes |
| TMIN | 0.179 | 0.096 | 1.87 | 0.03068 | no |
|  | 0.292 | 0.089 | 3.28 | 0.00051 | yes |

Standardised regression coefficients (beta) are shown from univariate regression and from multiple regression with model averaging to account for effects of body mass, habitat and migration.
