# Supplementary material for: An Indicator of the Impact of Climatic Change on European Bird Populations
Source: PLoS One. 2009 Mar 4;4(3):e4678. doi: 10.1371/journal.pone.0004678 (PMC2649536; doi:10.1371/journal.pone.0004678)
Supplement: Table S5 — Relationships between European bird species' trends, CRPs and body mass, controlling for the effects of phylogeny using a method of independent contrasts. (0.03 MB DOC) [file pone.0004678.s012.doc]

Table S5. Relationships between European bird species’ trends, CRPs and body mass, controlling for the effects of phylogeny using a method of independent contrasts.

| **Variable** | **Coeff.** | **SE** | ***t*** | ***P*** | **Controlled variable** |
| --- | --- | --- | --- | --- | --- |
| CLIMHaA2 | **0.013** | **0.007** | **2.01** | **0.024** | None |
|  | **0.015** | **0.006** | **2.24** | **0.014** | Body mass |
| CLIMHaB2 | **0.020** | **0.010** | **2.20** | **0.015** | None |
|  | **0.022** | **0.009** | **2.41** | **0.009** | Body mass |
| CLIMEcA2 | **0.013** | **0.007** | **1.93** | **0.029** | None |
|  | **0.014** | **0.007** | **2.14** | **0.017** | Body mass |
| CLIMEcB2 | *0.011* | *0.008* | *1.42* | *0.080* | None |
|  | **0.014** | **0.008** | **1.78** | **0.039** | Body mass |
| CLIMGfA2 | **0.040** | **0.012** | **3.27** | **0.001** | None |
|  | **0.039** | **0.012** | **3.22** | **0.001** | Body mass |
| CLIMGfB2 | **0.040** | **0.015** | **2.70** | **0.004** | None |
|  | **0.036** | **0.015** | **2.49** | **0.007** | Body mass |
| CLIMEns | **0.021** | **0.009** | **2.19** | **0.016** | None |
|  | **0.022** | **0.009** | **2.41** | **0.009** | Body mass |
| CST | 0.249 | 0.216 | 1.16 | 0.126 | None |
|  | 0.272 | 0.210 | 1.30 | 0.099 | Body mass |
| LAT | **-0.001** | **0.001** | **-1.79** | **0.038** | None |
|  | *-0.001* | *0.001* | *-1.56* | *0.061* | Body mass |
| TMEAN | **0.004** | **0.002** | **2.03** | **0.023** | None |
|  | **0.004** | **0.002** | **1.83** | **0.035** | Body mass |
| TMAX | *0.003* | *0.002* | *1.65* | *0.051* | None |
|  | **0.003** | **0.002** | **1.74** | **0.043** | Body mass |
| TMIN | **0.002** | **0.001** | **1.89** | **0.031** | None |
|  | *0.002* | *0.001* | *1.42* | *0.080* | Body mass |
| Body mass | **0.012** | **0.004** | **2.69** | **0.004** | - |

Contrasts were obtained using the program *Comparative analysis by independent contrasts* (CAIC, version 2·6·9 [37]) with the assumption that all branch lengths were equal. The table presents the results of regression analysis through the origin for each variable in turn (d.f.=1, 103), then controlling for the potential confounding effects of body mass (d.f.=2, 103). Values in bold are statistically significant with one-tailed *P*<0.05, those in italic *P*<0.1.
