# Supplementary material for: An Indicator of the Impact of Climatic Change on European Bird Populations
Source: PLoS One. 2009 Mar 4;4(3):e4678. doi: 10.1371/journal.pone.0004678 (PMC2649536; doi:10.1371/journal.pone.0004678)
Supplement: Table S6 — Interspecific Pearson correlation coefficients among CRPs. (0.03 MB DOC) [file pone.0004678.s013.doc]

Table S6. Interspecific Pearson correlation coefficients among CRPs.

|  | **CLIMHaA2** | **CLIMGfA2** | **CLIMEcB2** | **CLIMHaB2** | **CLIMGfB2** | **LAT** | **TMEAN** | **TMAX** | **TMIN** | **CST** |
| --- | --- | --- | --- | --- | --- | --- | --- | --- | --- | --- |
| **CLIMEcA2** | 0.958 | 0.828 | 0.973 | 0.911 | 0.762 | -0.842 | 0.845 | 0.806 | 0.616 | 0.608 |
| **CLIMHaA2** |  | 0.874 | 0.940 | 0.967 | 0.830 | -0.882 | 0.896 | 0.838 | 0.689 | 0.604 |
| **CLIMGfA2** |  |  | 0.795 | 0.898 | 0.964 | -0.755 | 0.773 | 0.673 | 0.668 | 0.543 |
| **CLIMEcB2** |  |  |  | 0.898 | 0.730 | -0.813 | 0.808 | 0.785 | 0.563 | 0.589 |
| **CLIMHaB2** |  |  |  |  | 0.852 | -0.851 | 0.874 | 0.768 | 0.720 | 0.524 |
| **CLIMGfB2** |  |  |  |  |  | -0.759 | 0.769 | 0.676 | 0.675 | 0.523 |
| **LAT** |  |  |  |  |  |  | -0.953 | -0.809 | -0.813 | -0.602 |
| **TMEAN** |  |  |  |  |  |  |  | 0.838 | 0.779 | 0.516 |
| **TMAX** |  |  |  |  |  |  |  |  | 0.514 | 0.554 |
| **TMIN** |  |  |  |  |  |  |  |  |  | 0.351 |

Results are for the 108 species included in the regression analyses.
