# Supplementary material for: An Indicator of the Impact of Climatic Change on European Bird Populations
Source: PLoS One. 2009 Mar 4;4(3):e4678. doi: 10.1371/journal.pone.0004678 (PMC2649536; doi:10.1371/journal.pone.0004678)
Supplement: Table S7 — Comparison of separate regressions of population trend on CLIM variables for species with negative values of the CLIM variable (CLIM−) and those with positive values of the CLIM variable (CLIM+). (0.04 MB DOC) [file pone.0004678.s014.doc]

Table S7. Comparison of separate regressions of population trend on CLIM variables for species with negative values of the CLIM variable (CLIM-) and those with positive values of the CLIM variable (CLIM+).

|  | **CLIM- species** | | | | **CLIM+ species** | | | | **pos. v. neg. *P*** |
| --- | --- | --- | --- | --- | --- | --- | --- | --- | --- |
| **CLIM** | **Beta** | **SE** | ***t*** | ***P*** | **Beta** | **SE** | ***t*** | ***P*** |  |
| CLIMEcA2 | 0.262 | 0.102 | 2.56 | 0.005 | 0.453 | 0.230 | 1.97 | 0.025 | 0.219 |
|  | 0.232 | 0.104 | 2.22 | 0.013 | 0.335 | 0.219 | 1.53 | 0.063 | 0.188 |
| CLIMHaA2 | 0.203 | 0.110 | 1.85 | 0.032 | 0.262 | 0.193 | 1.36 | 0.088 | 0.405 |
|  | 0.202 | 0.108 | 1.88 | 0.030 | 0.179 | 0.117 | 1.53 | 0.064 | 0.452 |
| CLIMGfA2 | 0.206 | 0.112 | 1.83 | 0.034 | 0.394 | 0.174 | 2.27 | 0.012 | 0.066 |
|  | 0.211 | 0.109 | 1.93 | 0.027 | 0.159 | 0.117 | 1.36 | 0.087 | 0.028 |
| CLIMEcB2 | 0.140 | 0.106 | 1.32 | 0.093 | 0.426 | 0.219 | 1.94 | 0.026 | 0.117 |
|  | 0.129 | 0.107 | 1.20 | 0.114 | 0.256 | 0.157 | 1.62 | 0.052 | 0.087 |
| CLIMHaB2 | 0.260 | 0.109 | 2.37 | 0.009 | 0.442 | 0.176 | 2.51 | 0.006 | 0.166 |
|  | 0.226 | 0.110 | 2.06 | 0.020 | 0.421 | 0.188 | 2.24 | 0.013 | 0.228 |
| CLIMGfB2 | 0.218 | 0.120 | 1.82 | 0.034 | 0.326 | 0.153 | 2.12 | 0.017 | 0.188 |
|  | 0.229 | 0.119 | 1.92 | 0.027 | 0.361 | 0.147 | 2.46 | 0.007 | 0.032 |
| CLIMEns | 0.225 | 0.106 | 2.13 | 0.017 | 0.396 | 0.211 | 1.88 | 0.030 | 0.254 |
|  | 0.220 | 0.103 | 2.14 | 0.016 | 0.275 | 0.117 | 2.35 | 0.009 | 0.219 |

For each CLIM, the standardised regression coefficient (beta) is given for the univariate regression (upper row) and model averaged multiple regression with effects of body mass, habitat and migratory status accounted for (lower row). One-tailed *P* values are given for the effects of the CLIM. The right hand column gives the two-tailed *P* value for a test by piecewise regression of whether the population trend vs. CLIM relationship differs between CLIM- and CLIM+ species.
