# Supplementary material for: An Indicator of the Impact of Climatic Change on European Bird Populations
Source: PLoS One. 2009 Mar 4;4(3):e4678. doi: 10.1371/journal.pone.0004678 (PMC2649536; doi:10.1371/journal.pone.0004678)
Supplement: Table S8 — Comparison of the European treat status of all breeding species (n = 526) and those used in the indicator analyses above (n = 122). (0.02 MB DOC) [file pone.0004678.s015.doc]

**Table S8. Comparison of the European treat status of all breeding species (n=526) and those used in the indicator analyses above (n=122).**

The classification of European threat follows Ref [9].
