# Supplementary material for: An Indicator of the Impact of Climatic Change on European Bird Populations
Source: PLoS One. 2009 Mar 4;4(3):e4678. doi: 10.1371/journal.pone.0004678 (PMC2649536; doi:10.1371/journal.pone.0004678)
Supplement: Table S9 — AICc weights for multiple regression models of population trend on CRP variables. (0.07 MB DOC) [file pone.0004678.s016.doc]

**Table S9.** **AICc weights for multiple regression models of population trend on CRP variables.**

| **Model specification** | | | | | | | **AICc weights** | | | | | | | | | | |  |
| --- | --- | --- | --- | --- | --- | --- | --- | --- | --- | --- | --- | --- | --- | --- | --- | --- | --- | --- |
| LMS | HAB | MIG | LMS.HAB | LMS.MIG | HAB.MIG | LMS.HAB.MIG | CLIMEcA2 | CLIMHaA2 | CLIMGfA2 | CLIMEcB2 | CLIMHaB2 | CLIMGfB2 | CLIMEns | CST | LAT | TMEAN | TMAX | TMIN |
|  | X | X |  |  | X |  | 0.4492 | 0.5368 | 0.5877 | 0.4698 | 0.5521 | 0.6500 | 0.5023 | 0.0001 | 0.4519 | 0.5224 | 0.5730 | 0.6200 |
| X | X | X |  |  | X |  | 0.5036 | 0.4233 | 0.3803 | 0.4828 | 0.4114 | 0.3209 | 0.4568 | 0.0001 | 0.4947 | 0.4289 | 0.3799 | 0.3412 |
| X | X | X |  | X | X |  | 0.0359 | 0.0308 | 0.0249 | 0.0357 | 0.0286 | 0.0231 | 0.0316 | 0.0008 | 0.0422 | 0.0396 | 0.0371 | 0.0274 |
| X | X | X | X |  | X |  | 0.0075 | 0.0064 | 0.0061 | 0.0073 | 0.0062 | 0.0055 | 0.0068 | 0.0000 | 0.0096 | 0.0076 | 0.0064 | 0.0105 |
| X | X |  |  |  |  |  | 0.0015 | 0.0009 | 0.0002 | 0.0017 | 0.0004 | 0.0001 | 0.0008 | 0.0001 | 0.0004 | 0.0003 | 0.0011 | 0.0001 |
|  | X |  |  |  |  |  | 0.0009 | 0.0007 | 0.0002 | 0.0010 | 0.0003 | 0.0001 | 0.0005 | 0.0000 | 0.0002 | 0.0002 | 0.0011 | 0.0001 |
| X | X | X | X | X | X |  | 0.0004 | 0.0004 | 0.0003 | 0.0005 | 0.0004 | 0.0003 | 0.0004 | 0.0015 | 0.0007 | 0.0006 | 0.0005 | 0.0006 |
| X | X | X |  |  |  |  | 0.0004 | 0.0003 | 0.0001 | 0.0004 | 0.0002 | 0.0000 | 0.0003 | 0.0002 | 0.0001 | 0.0001 | 0.0002 | 0.0000 |
|  | X | X |  |  |  |  | 0.0002 | 0.0002 | 0.0001 | 0.0002 | 0.0002 | 0.0001 | 0.0002 | 0.0001 | 0.0001 | 0.0001 | 0.0003 | 0.0000 |
| X | X |  | X |  |  |  | 0.0001 | 0.0001 | 0.0000 | 0.0001 | 0.0000 | 0.0000 | 0.0001 | 0.0000 | 0.0000 | 0.0000 | 0.0001 | 0.0000 |
| X | X | X | X | X | X | X | 0.0000 | 0.0000 | 0.0000 | 0.0000 | 0.0000 | 0.0000 | 0.0000 | 0.4252 | 0.0001 | 0.0000 | 0.0000 | 0.0001 |
|  |  |  |  |  |  |  | 0.0001 | 0.0001 | 0.0000 | 0.0001 | 0.0000 | 0.0000 | 0.0000 | 0.0000 | 0.0000 | 0.0000 | 0.0002 | 0.0000 |
| X | X | X |  | X |  |  | 0.0000 | 0.0000 | 0.0000 | 0.0000 | 0.0000 | 0.0000 | 0.0000 | 0.0000 | 0.0000 | 0.0000 | 0.0000 | 0.0000 |
| X |  |  |  |  |  |  | 0.0001 | 0.0000 | 0.0000 | 0.0001 | 0.0000 | 0.0000 | 0.0000 | 0.5091 | 0.0000 | 0.0000 | 0.0001 | 0.0000 |
| X | X | X | X |  |  |  | 0.0000 | 0.0000 | 0.0000 | 0.0000 | 0.0000 | 0.0000 | 0.0000 | 0.0528 | 0.0000 | 0.0000 | 0.0000 | 0.0000 |
|  |  | X |  |  |  |  | 0.0000 | 0.0000 | 0.0000 | 0.0000 | 0.0000 | 0.0000 | 0.0000 | 0.0089 | 0.0000 | 0.0000 | 0.0000 | 0.0000 |
| X |  | X |  |  |  |  | 0.0000 | 0.0000 | 0.0000 | 0.0000 | 0.0000 | 0.0000 | 0.0000 | 0.0000 | 0.0000 | 0.0000 | 0.0000 | 0.0000 |
| X | X | X | X | X |  |  | 0.0000 | 0.0000 | 0.0000 | 0.0000 | 0.0000 | 0.0000 | 0.0000 | 0.0009 | 0.0000 | 0.0000 | 0.0000 | 0.0000 |
| X |  | X |  | X |  |  | 0.0000 | 0.0000 | 0.0000 | 0.0000 | 0.0000 | 0.0000 | 0.0000 | 0.0002 | 0.0000 | 0.0000 | 0.0000 | 0.0000 |

All models include the effect of the single CRP indicated by the column headers CLIMEcA2 and headers to the right of it. The models also include the effects of log body mass (LMS), breeding habitat (HAB) and migratory status (MIG) in all possible combinations, including two- and three-way interactions. The variables and interactions included are indicated by the left-hand set of columns. Models are ranked from highest to lowest according to AICc weight for the models where CLIMEns was the CRP.
