# Supplementary material for: An Indicator of the Impact of Climatic Change on European Bird Populations
Source: PLoS One. 2009 Mar 4;4(3):e4678. doi: 10.1371/journal.pone.0004678 (PMC2649536; doi:10.1371/journal.pone.0004678)
Supplement: Table S10 — Estimates of parameters of two-period piecewise ordinary least squares regression models relating annual values of dependent variables to time (calendar year A.D.). (0.02 MB DOC) [file pone.0004678.s017.doc]

Table S10. Estimates of parameters of two-period piecewise ordinary least squares regression models relating annual values of dependent variables to time (calendar year A.D.).

| **Dependent variable** | ***b_0_*** | | ***b_1_*** | | ***b_2_*** | | ***t’*** | |
| --- | --- | --- | --- | --- | --- | --- | --- | --- |
| CII | 48.746 | (21.483) | 0.02467 | (0.01083) | 0.0274 | (0.0029) | 1986.19 | (1.12) |
| Climate | -0.190 | (176.12) | -0.00019 | (0.12738) | 0.0844 | (0.0246) | 1985.00 | (2.51) |

The dependent variables are: (1) the Climate Impact Index (CII) for 1980 – 2005; and (2) all three of the climate variables GDD5, MTCO and MTEMP for 1980 - 2002. CII was expressed as a proportion of the 1980 index value and log-transformed before analysis; climate variables were standardised but not log-transformed. Asymptotic standard errors are given in parentheses.
