# Supplementary material for: An Indicator of the Impact of Climatic Change on European Bird Populations
Source: PLoS One. 2009 Mar 4;4(3):e4678. doi: 10.1371/journal.pone.0004678 (PMC2649536; doi:10.1371/journal.pone.0004678)
Supplement: Table S11 — Tests of whether the slope of the relationship between population trend and CLIMEns varies significantly among classes of breeding habitat (HAB) or migratory status (MIG), with log body mass (LMS), or with the goodness-of-fit of the climate envelope model used to calculate CLIMEns (AUC). (0.02 MB DOC) [file pone.0004678.s018.doc]

Table S11. Tests of whether the slope of the relationship between population trend and CLIMEns varies significantly among classes of breeding habitat (HAB) or migratory status (MIG), with log body mass (LMS), or with the goodness-of-fit of the climate envelope model used to calculate CLIMEns (AUC).

| **Base model** | **Interaction with CLIMEns** | **d.f.** | ***F*** | ***P*** |
| --- | --- | --- | --- | --- |
| CLIMEns+HAB+MIG+HAB.MIG | HAB | 3, 92 | 0.32 | 0.813 |
| CLIMEns+HAB+MIG+HAB.MIG | MIG | 2, 93 | 1.33 | 0.269 |
| CLIMEns+HAB+MIG+HAB.MIG | AUC | 2, 93 | 0.24 | 0.787 |
| CLIMEns+LMS+HAB+MIG+HAB.MIG | LMS | 1, 93 | 0.49 | 0.484 |
| CLIMEns+LMS+HAB+MIG+HAB.MIG | HAB | 3, 91 | 0.23 | 0.878 |
| CLIMEns+LMS+HAB+MIG+HAB.MIG | MIG | 2, 92 | 1.63 | 0.202 |
| CLIMEns+LMS+HAB+MIG+HAB.MIG | AUC | 2, 92 | 0.15 | 0.861 |

The interactions are tested by adding them to two base models with the highest level of support. The test of AUC is that of the main effect and AUC.CLIMEns interaction together.
