# Supplementary material for: An Indicator of the Impact of Climatic Change on European Bird Populations
Source: PLoS One. 2009 Mar 4;4(3):e4678. doi: 10.1371/journal.pone.0004678 (PMC2649536; doi:10.1371/journal.pone.0004678)
Supplement: Table S12 — Comparison of separate regressions of population trend on CRPs for species with or without part of their breeding range in North Africa. (0.03 MB DOC) [file pone.0004678.s019.doc]

Table S12. Comparison of separate regressions of population trend on CRPs for species with or without part of their breeding range in North Africa.

|  | **Non- North African species** | | | | **North African species** | | | | **interaction *P*** |
| --- | --- | --- | --- | --- | --- | --- | --- | --- | --- |
| **CRP** | **Beta** | **SE** | ***t*** | ***P*** | **Beta** | **SE** | ***t*** | ***P*** |  |
| CLIMEcA2 | 0.088 | 0.146 | 0.60 | 0.276 | 0.227 | 0.129 | 1.76 | 0.042 | 0.350 |
| CLIMHaA2 | 0.160 | 0.144 | 1.11 | 0.137 | 0.231 | 0.129 | 1.79 | 0.039 | 0.338 |
| CLIMGfA2 | 0.303 | 0.139 | 2.18 | 0.017 | 0.305 | 0.126 | 2.42 | 0.009 | 0.272 |
| CLIMEcB2 | 0.015 | 0.157 | 0.09 | 0.463 | 0.191 | 0.130 | 1.47 | 0.074 | 0.293 |
| CLIMHaB2 | 0.175 | 0.143 | 1.22 | 0.114 | 0.239 | 0.129 | 1.86 | 0.034 | 0.348 |
| CLIMGfB2 | 0.270 | 0.140 | 1.93 | 0.030 | 0.243 | 0.128 | 1.89 | 0.032 | 0.345 |
| CLIMEns | 0.166 | 0.144 | 1.15 | 0.127 | 0.244 | 0.128 | 1.90 | 0.031 | 0.352 |

For each CRP, the standardised regression coefficient (beta) is given for the univariate regression. One-tailed *P* values are given for the effects of the CRPs. The right-hand column gives the two-tailed *P* value for a test of the two-way interaction term which tests for a difference in the slope of the population trend between species with and without part of their breeding range in North Africa.
